# Mitochondrial dysfunction, metabolic quiescence and premature senescence in CMT4B3 fibroblasts

**DOI:** 10.1093/hmg/ddag080

**Published:** 2026-08-21

**Authors:** Paola Zanfardino, Alessandro Amati, Sharon Cox, Gennaro Agrimi, Stefano Doccini, Patricio Fernández-Silva, Apollonia Tullo, Jessica Rosati, Filippo M Santorelli, Vittoria Petruzzella

**Affiliations:** Department of Translational Biomedicine and Neuroscience - DiBraiN, University of Bari Aldo Moro, Piazza Giulio Cesare 11, 70124, Bari, Italy; Department of Translational Biomedicine and Neuroscience - DiBraiN, University of Bari Aldo Moro, Piazza Giulio Cesare 11, 70124, Bari, Italy; Department of Biosciences, Biotechnology and Environment, University of Bari Aldo Moro, Via Orabona, 70124 Bari, Italy; Department of Biosciences, Biotechnology and Environment, University of Bari Aldo Moro, Via Orabona, 70124 Bari, Italy; U.O.C. Medicina Molecolare, Neurogenetica e Malattie Neuromuscolari IRCCS Fondazione Stella Maris, Viale del Tirreno, 156018 CALAMBRONE, Pisa, Italy; Department Biochemistry and Molecular and Cell Biology, Faculty of Sciences, Campus San Francisco Square, Biocomputation and Complex Systems Physics Institute (BIFI), University of Zaragoza, E-50009 Zaragoza, Spain, Zaragoza, Spain; Institute of Biomembrane, Bioenergetics and Molecular Biotechnology (IBIOM) –Bari - CNR, -Via Amendola 122/D 70126, Bari, Italy; Cellular Reprogramming Unit, Fondazione IRCCS Casa Sollievo della Sofferenza, 71013, San Giovanni Rotondo, Italy; U.O.C. Medicina Molecolare, Neurogenetica e Malattie Neuromuscolari IRCCS Fondazione Stella Maris, Viale del Tirreno, 156018 CALAMBRONE, Pisa, Italy; Department of Translational Biomedicine and Neuroscience - DiBraiN, University of Bari Aldo Moro, Piazza Giulio Cesare 11, 70124, Bari, Italy

**Keywords:** MTMR5/SBF1, mitochondria, oxidative phosphorylation, transcriptomics, senescence, Charcot–Marie–tooth disease type 4B3

## Abstract

Charcot–Marie–Tooth disease type 4B3 (CMT4B3) is an ultra-rare autosomal recessive neuropathy caused by mutations in the MTMR5/SBF1 gene. In this study, we characterized dermal fibroblasts derived from a patient carrying compound-heterozygous MTMR5/SBF1 variants (R763H/G1064E) and identified alterations affecting mitochondrial metabolism and cellular stress pathways. Patient fibroblasts exhibited fragmented mitochondrial networks with a shift toward fission, together with reduced ATP production, while mitochondrial mass, respiratory chain assembly, and markers of mitochondrial biogenesis were preserved. In line with our previous evidence of enhanced mitophagy, these findings support the presence of altered mitochondrial quality control. The reduction in cellular energy production was not accompanied by increased glycolytic activity, indicating a metabolically quiescent phenotype. Transcriptomic profiling revealed dysregulation of the PI3K/AKT signalling pathway. AKT phosphorylation at Ser473 was increased in the absence of complete canonical AKT activation. These signalling changes were associated with increased expression of p53 and p21 and with features consistent with premature cellular senescence. Overall, our findings identify metabolic quiescence and premature senescence as previously unrecognized aspects of CMT4B3 cellular pathology and suggest that altered coordination between mitochondrial metabolism and intracellular signalling may contribute to disease pathogenesis.

## Graphical Abstract

**Graphical Abstract:**
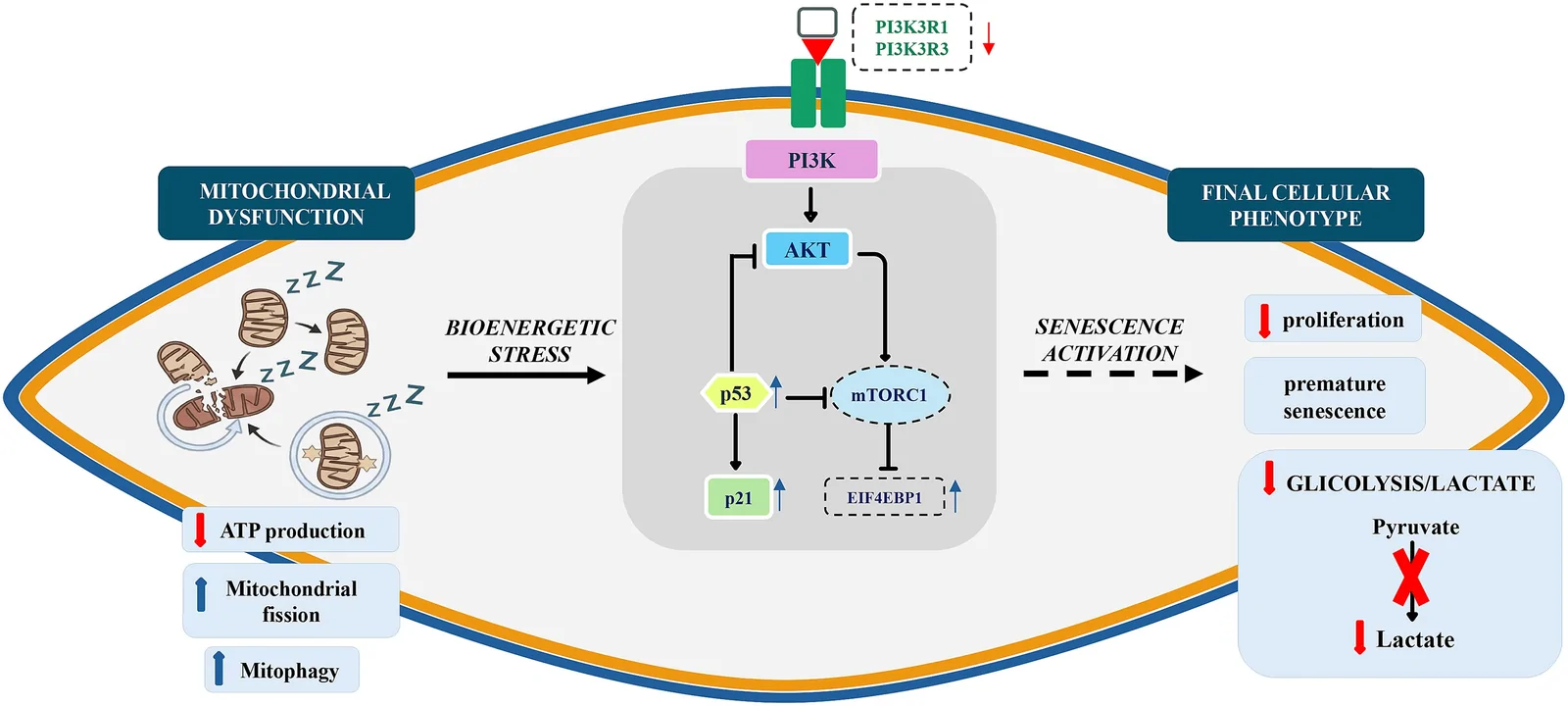

## Introduction

Charcot–Marie-Tooth (CMT) disease represents a clinically and genetically heterogeneous group of inherited peripheral neuropathies characterized by progressive distal muscle weakness, sensory impairment, and variable electrophysiological features [1–3]. Charcot–Marie–Tooth disease type 4B3 (CMT4B3) is an ultra-rare autosomal recessive neuropathy caused by mutations in the MTMR5^*^gene, also known as *SET Binding Factor* 1 (*SBF*1). The human MTMR family consists of 16 paralogs, including *MTM1* and *MTMR1–MTMR13* [4]. Among them, nine function as catalytically active phosphatases, whereas the remaining seven are pseudophosphatases lacking enzymatic activity [5, 6]. MTMR proteins regulate phosphoinositide turnover and participate in several cellular processes, including membrane trafficking, cytoskeletal organisation, membrane remodelling, autophagy, and intracellular signalling pathways [7–13]. Although catalytically inactive, MTMR5/SBF1 forms heterodimers with the active phosphatase MTMR2, modulating its localisation and enhancing its activity [14, 15]. Genetic studies in mouse models have shown that MTMR5/SBF1 plays a specific and non-redundant role in peripheral nerve development, distinct from MTMR13/SBF2, supporting a broader involvement of MTMR proteins in nerve biology [16]. MTMR5/SBF1 functionally interacts with MTMR2 and MTMR13/SBF2 in pathways required for peripheral nerve maintenance, and disruption of this network contributes to the development of CMT4B3 (OMIM:615284) [17]. CMT4B3 is considered an exceptionally rare condition and is clinically characterised by an early-onset, slowly progressive sensorimotor neuropathy with both axonal and demyelinating features [2, 17]. Although only a limited number of families carrying pathogenic *MTMR5/SBF1* variants have been identified to date [17–23], recent studies have expanded the phenotypic spectrum of CMT4B3, revealing marked variability in clinical presentation and disease severity and highlighting the complexity of genotype–phenotype correlations in *MTMR5/SBF1*-related neuropathy [2, 24]. Although *MTMR5/SBF1* has been identified as the causative gene for CMT4B3, the cellular mechanisms underlying disease pathogenesis remain poorly understood. Owing to the rarity of the disease, genotype–phenotype relationships are incompletely defined, and the downstream consequences of MTMR5 dysfunction on cellular homeostasis remain largely unclear. To further investigate the pathogenic effects of *MTMR5/SBF1* dysfunction, we extended the functional characterisation of a previously reported *MTMR5/SBF1* variant [23] in patient-derived dermal fibroblasts, a well-established human cellular model for studying disease-related alterations in energy metabolism, mitochondrial homeostasis, and stress-response signalling [25–28].

## Results

### Case report and fibroblast-based experimental model

The case of CMT4B3 reported by Berti et al. in 2021 described a 12-year-old Italian girl with no relevant family or prenatal history. She exhibited neuromotor developmental delay starting at 18 months, accompanied by muscle weakness, respiratory difficulties, and moderate to severe axonal motor polyneuropathy, despite normal nerve conduction velocity (NCV). Electroneurography revealed diffuse reduction of compound muscle action potentials (CMAPs, values < 1.5 mV), confirming the predominance of axonal involvement [23]. Genetic analysis identified two biallelic mutations in the MTMR5/SBF1 gene (c.2288G > A/p.Arg763His and c.3194G > A/p.Gly1064Glu), confirming a diagnosis of CMT4B3. A skeletal muscle biopsy showed histopathological features consistent with chronic neurogenic damage, including extensive homotypic fibre-type grouping on ATPase staining, particularly involving type I fibres. NADH staining revealed marked disorganisation of the intermyofibrillar network in nearly all type I fibres, with lobulated fibres, focal core-like areas and irregular oxidative enzyme activity, indicating structural abnormalities of the muscle fibres. No SDH overexpression or abnormal glycogen or lipid accumulation was observed [23].

For our study, we used sex- and age-matched controls alongside patient-derived fibroblasts. Control fibroblasts included healthy skin-derived cells from individuals without any clinical or biochemical evidence of mitochondrial disease (Controls 1 and 2), as well as commercially available Normal Human Dermal Fibroblasts (NHDF, Control 3). All three controls were utilized for structural and functional mitochondrial analyses. However, for experiments requiring larger cell numbers, such as Blue Native PAGE (BN-PAGE), we limited controls to two due to the practical challenges of expanding primary fibroblast cultures. For Seahorse metabolic assays, we specifically selected and used control fibroblasts (*Control 1*) obtained through the same skin biopsy isolation and culture procedures as the patient’s fibroblasts. This approach ensured consistency in sample handling and minimized variability due to differences in cell origin or processing. To further reduce potential artefacts related to cellular senescence, all experiments were performed on cells with comparable passage numbers, ranging from passages 8 to 12.

### Altered mitochondrial dynamics in CMT4B3 fibroblasts: Normal fusion protein levels and increased response to fission inhibition

Abnormal mitochondrial function has been reported in CMT4B3 patient-derived cells [23, 28], and our recent study reported increased mitophagy in these cells [27]. To further characterize mitochondrial alterations, we analyzed mitochondrial morphology in CMT4B3 fibroblasts cultured under basal (glucose) and oxidative phosphorylation (OxPhos)-dependent (galactose) conditions using immunofluorescence with an anti-porin antibody. Compared to controls, CMT4B3 fibroblasts displayed significant morphological abnormalities: under basal conditions, there was an increased proportion of intermediate (~33%) and fragmented mitochondria (~37%), coupled with a reduction in tubular forms (~26%) (Fig. 1a). Mitochondrial fragmentation was further enhanced upon CCCP-induced uncoupling in glucose medium (~36%), with only partial recovery post-washout, as intermediate forms remained elevated (~40%). Under OxPhos-reliant conditions (galactose), fragmented and intermediate mitochondria were consistently increased, indicating more severe mitochondrial impairment.

**Figure 1:**
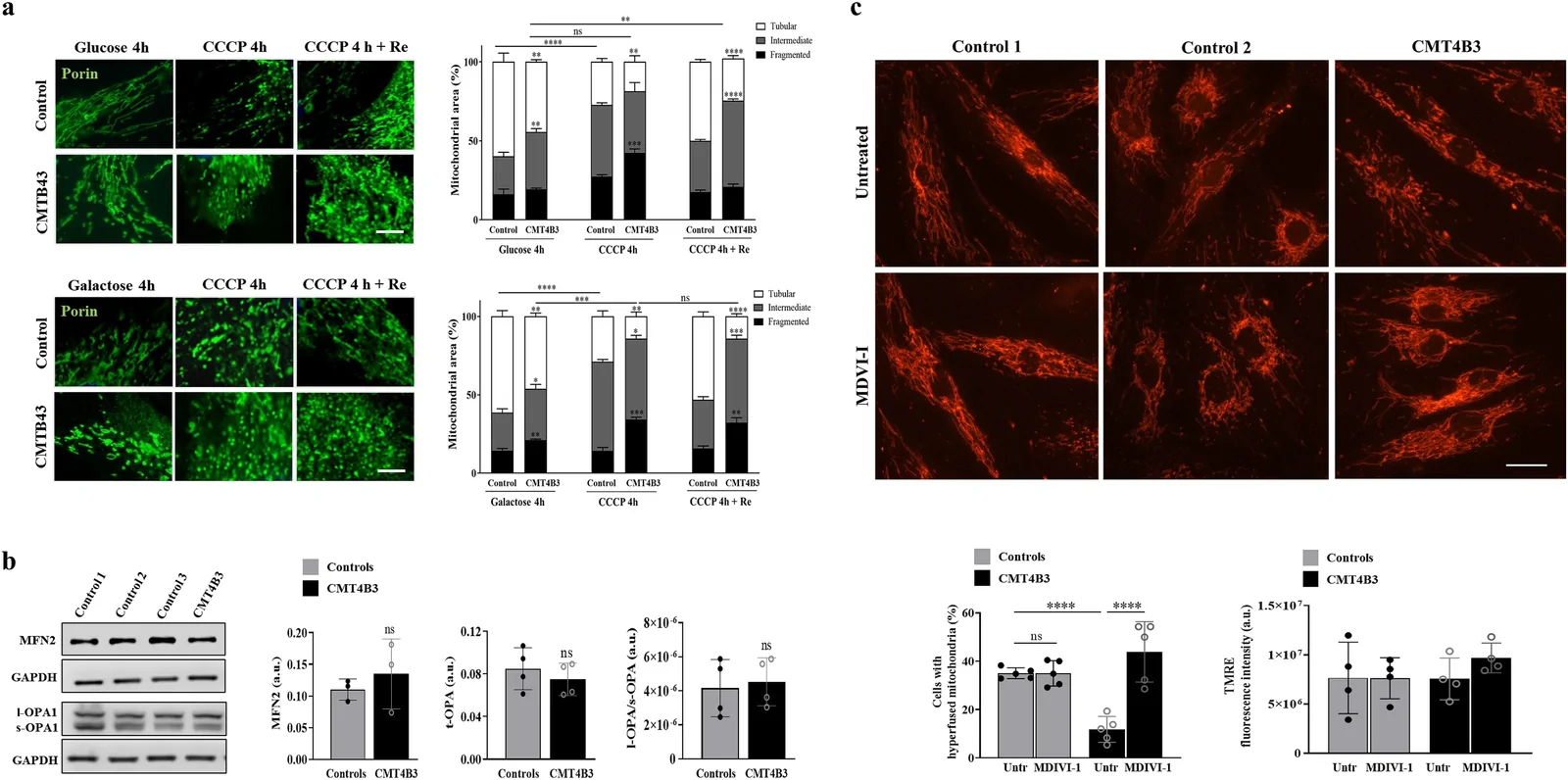
Mitochondrial network morphology is impaired in CMT4B3 fibroblasts despite normal levels of mitochondrial fusion proteins. (a) Representative immunofluorescence images of the mitochondrial network in control and CMT4B3 fibroblasts cultured in glucose or galactose medium. Cells were treated for 4 hours with 10 μM CCCP (CCCP 4 h) or with CCCP followed by a 4-hour recovery in the initial medium (CCCP 4 h + Re). Mitochondria were visualized by immunostaining with anti-VDAC-1/porin antibody. Scale bars: 25 μm. The bar graphs show the distribution of mitochondrial morphology (tubular, intermediate, fragmented) in fibroblasts under each condition. (b) Western blot analysis of mitochondrial fusion proteins MFN2 and OPA1 in total lysates from control and CMT4B3 fibroblasts. Densitometric quantification shows total OPA1 (long and short isoforms), the l-OPA1/s-OPA1 ratio, and MFN2 levels normalized to GAPDH. (c) Representative images from live-cell imaging of TMRE-stained control and CMT4B3 fibroblasts treated for 4 hours with 50 μM Mdivi-1. Scale bars: 25 μm. Data are presented as mean ± SD from at least three independent experiments (n ≥ 3). Statistical analysis was performed using Student’s *t-*test (^*^P < .05; ^**^P < .01; ^***^P < .001; ^****^P < .0001). For multiple comparisons, one-way ANOVA followed by Bonferroni’s post hoc test was used.

To assess whether increased fragmentation was due to impaired mitochondrial fusion, we measured the expression of key fusion proteins, MFN2 and OPA1, by Western blotting. Both steady-state levels and the long-to-short OPA1 isoform ratio (l-OPA1:s-OPA1) were comparable between CMT4B3 and control cells (Fig. 1b), suggesting fusion is preserved. We thus hypothesized that enhanced mitochondrial fission contributes to the phenotype. Treatment with Mdivi-1, a selective DRP1 inhibitor, induced significant mitochondrial elongation and interconnectivity in CMT4B3 fibroblasts, with a 2.73-fold increase in hyperfused mitochondria relative to basal conditions (Fig. 1c). Importantly, TMRE staining showed no changes in mitochondrial membrane potential after Mdivi-1 treatment, indicating that modulation of mitochondrial morphology by DRP1 inhibition occurs independently of membrane potential. These findings suggest heightened sensitivity of CMT4B3 mitochondria to DRP1-mediated fission modulation, underlying their altered morphology despite preserved fusion protein expression (Fig. 1c). Of note, although Mdivi-1 at 50 μM has been reported to exert off-target effects on Complex I [29], the preserved mitochondrial membrane potential and intact respiratory complex assembly observed in our BN-PAGE analysis are consistent with a morphological response driven primarily by DRP1 inhibition rather than by non-specific drug effects.

### Reduced total ATP steady-state levels in CMT4B3 fibroblasts

To investigate whether mitochondrial morphological alterations in CMT4B3 fibroblasts are associated with defects in mitochondrial function, we first assessed mitochondrial membrane potential (ΔΨm) using TMRE staining (Fig. 2a and b). Under basal conditions, ΔΨm appeared comparable between CMT4B3 and control fibroblasts, as indicated by TMRE fluorescence intensity (Figs. 1c and 2b). However, TMRE provides only a semiquantitative assessment of ΔΨm and does not allow precise quantification of membrane potential. Upon inhibition of ATP synthase with oligomycin, only control cells exhibited the expected mitochondrial hyperpolarisation (~25% increase after 15 minutes), whereas CMT4B3 fibroblasts showed no appreciable change in TMRE signal, suggesting defective maintenance or generation of the proton gradient under stress conditions (Fig. 2b).

**Figure 2:**
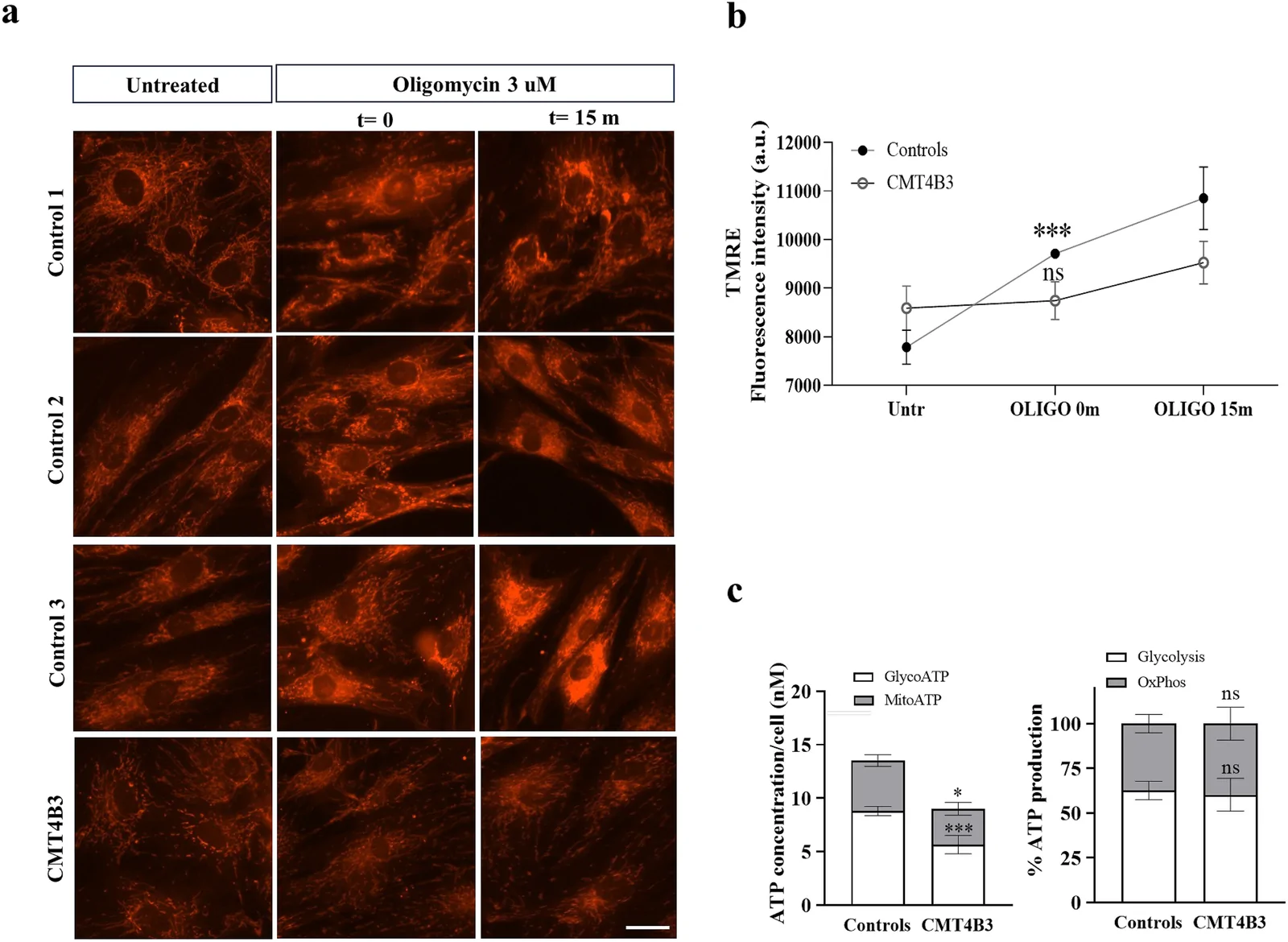
Total ATP steady-state levels are reduced in CMT4B3 fibroblasts. (a) Representative live-cell imaging of TMRE-stained control and CMT4B3 fibroblasts. Cells were treated with 3 μM oligomycin, and fluorescence intensity was assessed immediately and 15 minutes after treatment. Scale bars: 25 μm. (b) Quantification of TMRE fluorescence intensity in untreated cells (*Untr*), immediately after oligomycin addition, and 15 minutes post-treatment. Data corresponds to the experimental conditions shown in panel (a). (c) Quantification of total ATP levels and relative contributions from glycolysis and oxidative phosphorylation (OxPhos) in control and CMT4B3 fibroblasts. ATP production was assessed by luminescence using specific metabolic inhibitors, as detailed in the methods section. Data are expressed as mean ± SD from at least three independent experiments (n ≥ 3). Statistical significance was evaluated using Student’s *t-*test (^*^P < .05; ^**^P < .01; ^***^P < .001; ^****^P < .0001). For multiple comparisons, one-way ANOVA followed by Bonferroni’s post hoc test was applied.

After confirming cell viability by Trypan Blue staining, we next assessed total ATP content using a luminescence-based assay. CMT4B3 fibroblasts exhibited a significant reduction in ATP levels following inhibition of either OxPhos (~36%) or glycolysis (~29%) (Fig. 2c). In line with these findings, the total cellular ATP concentration per cell was markedly lower in CMT4B3 fibroblasts compared to controls (9.0 ± 0.7 nM vs. 13.5 ± 0.5 nM, respectively) (Fig. 2c). Despite this reduction, the proportional contributions of OxPhos and glycolysis to ATP production remained comparable between groups (Fig. 2c).

### Mitochondrial dysfunction and impaired ATP production in CMT4B3 fibroblasts

To further assess mitochondrial function, we performed polarographic oxygen measurement by Seahorse analyses. The Seahorse Mito Stress Test indicated broad mitochondrial dysfunction: basal OCR (~27.5%) (Fig. 3d), ATP-coupled OCR (~23.4%) (Fig. 3e), maximal respiration (~56%) (Fig. 3f), and spare respiratory capacity (~67.4%) (Fig. 3g) were significantly reduced in CMT4B3 cells. Despite this, mitochondrial membrane potential remained stable, likely due to a compensatory decrease in proton leak (~58.6%) (Fig. 3h).

**Figure 3:**
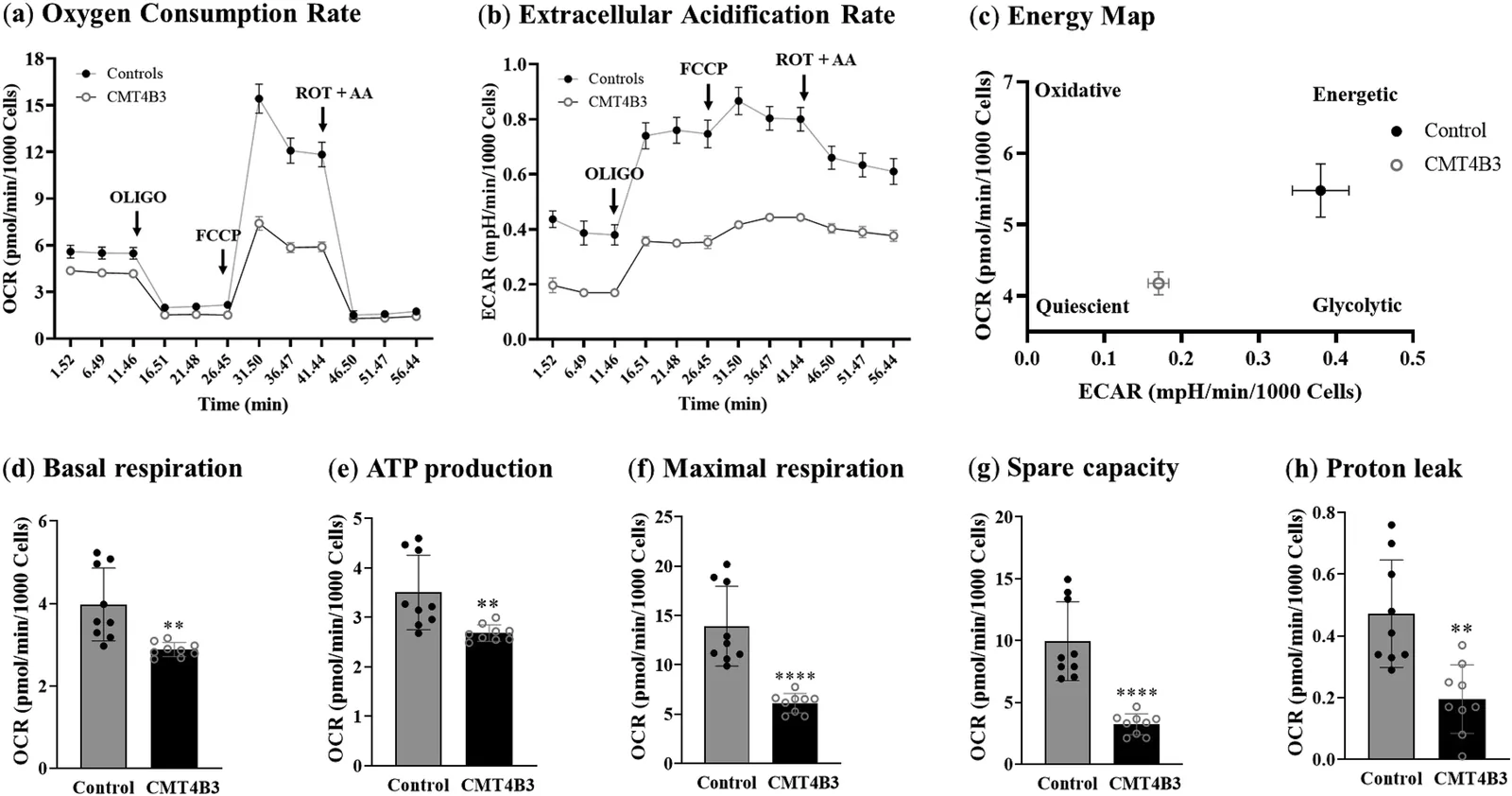
Mitochondrial respiratory capacity is reduced in CMT4B3 fibroblasts. (a, b) representative profiles of oxygen consumption rate (*OCR*) and extracellular acidification rate (*ECAR*) in control and CMT4B3 fibroblasts, measured under basal conditions and after sequential injection of oligomycin (*OLIGO*), *FCCP*, and rotenone plus antimycin a (*ROT + AA*). Data represents the mean ± SD of nine independent experiments. (c) Energy phenotype plot (OCR *vs* ECAR) under basal conditions in control and CMT4B3 fibroblasts. (d–h) Quantification of mitochondrial respiratory parameters: Basal respiration (d), ATP-linked OCR (e), maximal respiration (f), spare respiratory capacity (g), and proton leak (h). Data are expressed as mean ± SD from at least three independent experiments (n ≥ 3). Statistical significance was assessed using Student’s *t-*test (^*^P < .05; ^**^P < .01; ^***^P < .001; ^****^P < .0001).

To evaluate the functional consequences on energy production, total ATP content was also quantified using Seahorse-based methods. The Seahorse ATP Rate Assay provides a more accurate estimation of mitochondrial ATP production by converting ATP-coupled OCR values using theoretical P/O ratios, which account for the stoichiometry of ATP synthesis and F1F0-ATP synthase efficiency. The Seahorse Real-Time ATP Rate Assay revealed that OxPhos-derived ATP production rate was reduced by 27.9% and glycolytic ATP by 77% in CMT4B3 cells (Fig. 4d). Although the absolute values differed from those measured using the luminescence assay, both approaches consistently indicated impaired ATP production from both OxPhos and glycolysis. Notably, the relative contribution of each pathway to total ATP production remained comparable between CMT4B3 and control cells, as assessed by both luminescence and Seahorse essays (Figs. 2c and 4e). Consistently, lactate secretion (Fig. S1), OCR (Figs. 3a and 4a), and ECAR (Figs. 3b and 4b) were also reduced, positioning CMT4B3 fibroblasts within a metabolically quiescent region of the Seahorse energetic map, with globally diminished oxidative and glycolytic activity compared to the control group (Figs. 3c, 4c and f).

**Figure 4:**
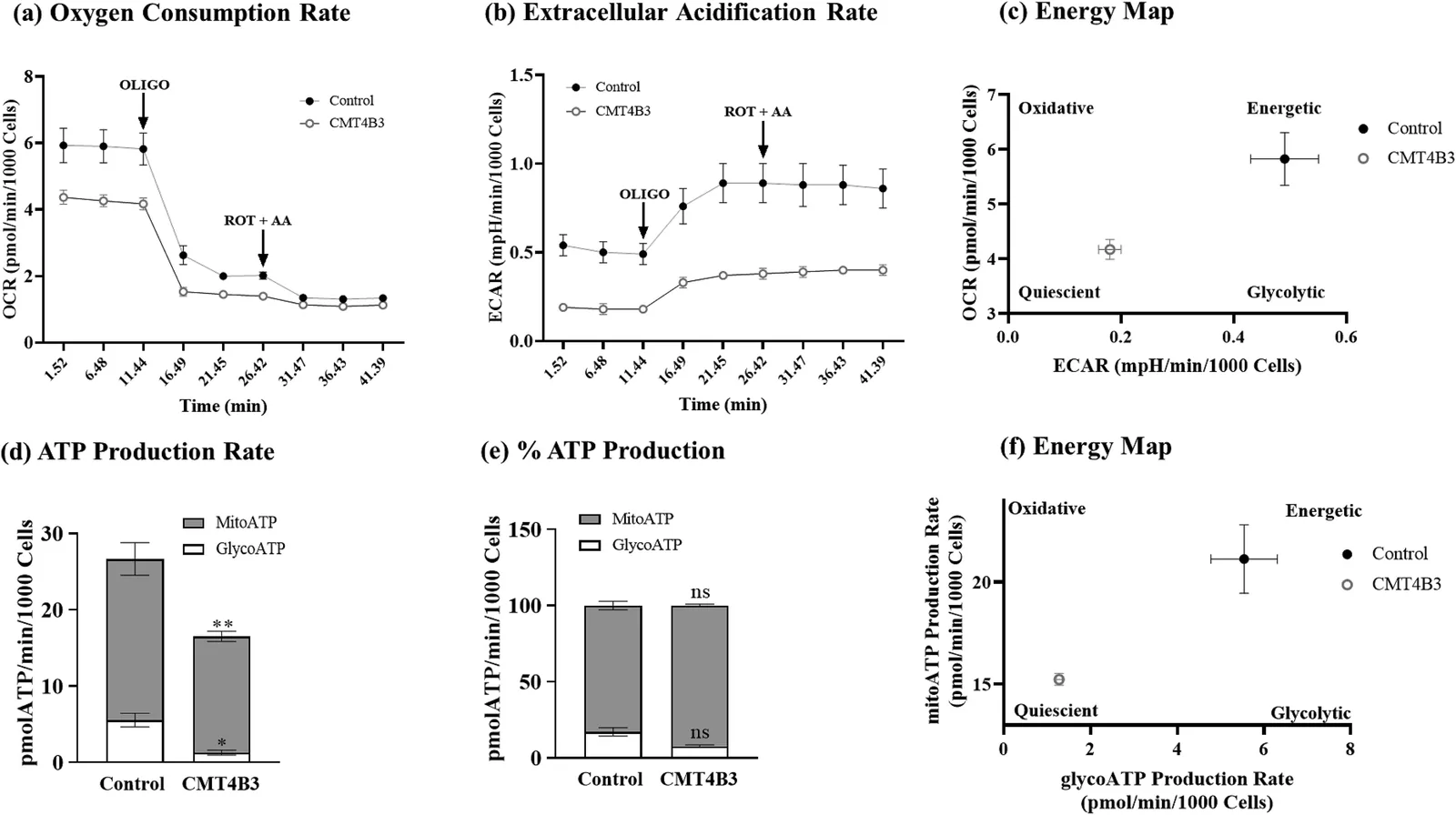
CMT4B3 fibroblasts exhibits a quiescent bioenergetic phenotype with reduced total ATP production. (a, b) representative profiles of oxygen consumption rate (*OCR*) and extracellular acidification rate (*ECAR*) in control and CMT4B3 fibroblasts, measured under basal conditions and after sequential injection of oligomycin (*OLIGO*) and rotenone plus antimycin a (*ROT + AA*). (c) Energy phenotype plot (OCR *vs* ECAR) under basal conditions for control and CMT4B3 fibroblasts. (d,e) Quantification of total ATP production rate (d) and the relative contribution of mitochondrial versus glycolytic ATP production (e). (f) Bioenergetic map showing mitochondrial ATP production rate versus glycolytic ATP production rate in control and CMT4B3 fibroblasts. Data is presented as mean ± SD from at least three independent experiments (n ≥ 3). Statistical significance was assessed using Student’s *t-*test (^*^P < .05; ^**^P < .01; ^***^P < .001; ^****^P < .0001).

### Unchanged ROS and superoxide levels in CMT4B3 fibroblasts

Given the significant mitochondrial dysfunction observed, we investigated the production of reactive oxygen species (ROS) and superoxide under both basal and stimulated conditions (Fig. S2). No differences were found in basal ROS or superoxide levels between CMT4B3 and control fibroblasts. However, after stimulation with pyocyanin, a known ROS inducer, both cell types displayed substantial increases in oxidative species: in control cells, ROS levels rose by 11.83-fold and superoxide levels increased by 5.65-fold. In CMT4B3 cells, ROS levels rose by 14.21-fold, while superoxide increased by 3.53-fold (Fig. S2). The greater ROS increase in CMT4B3 cells may reflect differential processing of superoxide into downstream ROS species, possibly due to altered redox buffering or antioxidant activity. These findings suggest that, despite impaired respiratory chain activity, ROS production remains tightly regulated under basal conditions.

### Structural integrity of mitochondrial respiratory complexes and of the genome is maintained in CMT4B3 fibroblasts

To determine whether structural alterations of the mitochondrial respiratory chain complexes could underlie the observed dysfunction in CMT4B3 fibroblasts, we investigated the organization and assembly of individual respiratory chain complexes and respiratory supercomplexes. BN-PAGE analysis of mitoplasts solubilized with DDM (Fig. S3a) revealed that complexes I, IV, and V were present and correctly assembled in both CMT4B3 and control fibroblasts. In parallel, digitonin-solubilized mitoplasts were used to preserve respiratory supercomplexes, showing comparable complex I-containing supercomplexes in both groups, while complex IV was mainly observed as a monomer, consistent with previous reports on fibroblast models [30, 31]. In-gel activity assays confirmed that these complexes were functionally active.

Steady-state levels of selected OxPhos subunits (mt-ND4, NDUFS1, COX IV, ATP5F1B) and the mitochondrial mass marker TOMM20 were unchanged between groups (Fig. S3b), indicating preserved respiratory chain composition and mitochondrial content.

Long-range PCR analysis revealed intact mitochondrial DNA in CMT4B3 fibroblasts, with no detectable deletions or rearrangements (Fig. S3c). Quantitative PCR showed no significant differences in mtDNA copy number (Fig. S3d). Lastly, expression levels of the transcription factors TFAM and TFEB, regulators of mitochondrial DNA maintenance and mitochondrial-lysosomal biogenesis, respectively, were comparable between patient and control cells (Fig. S3e), supporting unaltered mitochondrial biogenesis and transcriptional control in CMT4B3 fibroblasts.

### Transcriptome profiling reveals PI3K/AKT pathway involvement in CMT4B3 fibroblasts

RNA-seq analysis of CMT4B3 *versus* control fibroblasts identified 996 differentially expressed genes (DEGs) with GFOLD > 1.5. The full transcriptomic dataset is reported in Supplementary File S1. These DEGs were enriched in processes related to extracellular matrix (ECM) organization, cell adhesion, and signal transduction, with many localized to the extracellular region and annotated as ECM components or heparin-binding proteins (Table S1). KEGG pathway analysis highlighted enrichment in axon guidance, cytokine-cytokine receptor interaction, and notably the PI3K-AKT signalling pathway (Fig. S4, Table S2). Ingenuity Pathway Analysis (IPA) of the mTOR axis revealed dysregulation of key components: upregulation of REDD1/DDIT4, EIF4EBP1, RAC2, and PGF/VEGF; and downregulation of PIK3R1, PIK3R3, PP2A via its subunits PPM1L and PPP2R2E, RPS4Y1, and RPS9, suggesting alterations in mTOR-dependent translation (Fig. S5).

STRING-based protein–protein interaction (PPI) network analysis (791 nodes, 2753 edges) followed by MCODE clustering identified six dense modules (Fig. S6, Table S3). These were enriched in pathways such as ion transport (notably K^+^), immune response, MHC class I antigen presentation, Wnt signalling regulation, collagen organization, and metabolic processes, including monocarboxylic acid and alcohol metabolism.

Allelic expression analysis confirmed biallelic transcription of MTMR5/SBF1 mutations in patient fibroblasts. Mutant alleles were detected in ~ 47–48% of reads at both variant sites (Fig. S7), indicating likely co-expression of mutant and wild-type protein isoforms.

### The serine/threonine-protein kinase AKT is increasingly phosphorylated at Ser473 in CMT4B3 fibroblasts

Serine/threonine-protein kinase AKT displays increased phosphorylation at Ser473 as shown by immunoblotting analysis in CMT4B3 fibroblasts, which aligns with the enrichment of PI3K/AKT signalling observed in our transcriptomic analysis. This suggests that mitochondrial dysfunction is detected by cytosolic stress integrators such as AKT. Since AKT Ser473 phosphorylation is mediated by mTORC2 and can be triggered by energetic stress [32, 33], we investigated whether the energy metabolism defects in CMT4B3 fibroblasts were associated with altered AKT signalling. When PI3K is activated, AKT is first phosphorylated at Thr308 by PDK1, and is then fully activated by Ser473 phosphorylation through mTORC2 [34]. The activity of AKT depends on both phosphorylation sites, which reflects the functions of PDK1 and mTORC2 [35–37]. Our immunoblotting analysis showed a significant increase (~5.46-fold) in the AKT Ser473/total AKT *ratio* in CMT4B3 fibroblasts (Fig. 5a), while the Thr308/total AKT *ratio* remained unchanged.

**Figure 5:**
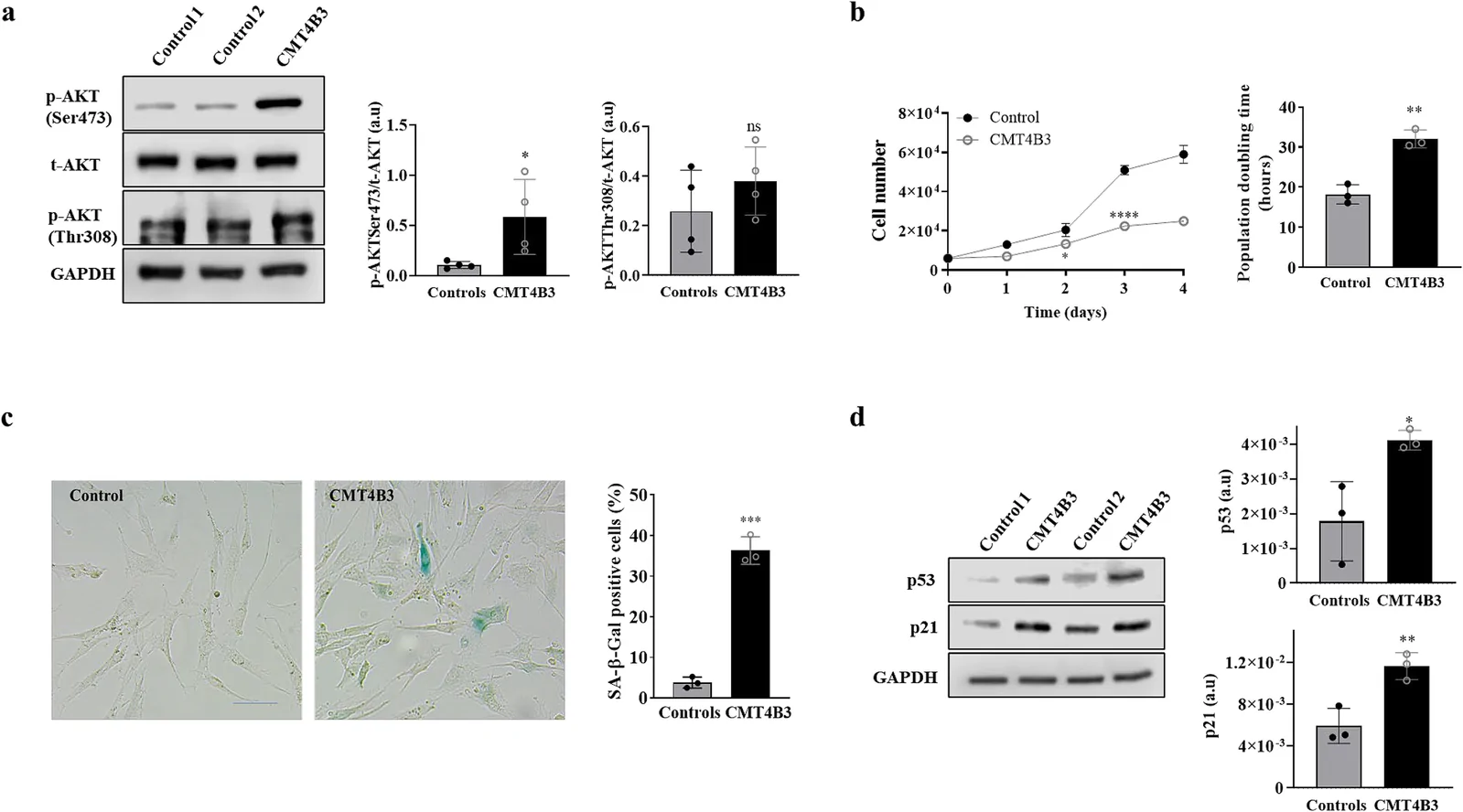
CMT4B3 fibroblasts display increased AKT phosphorylation, reduced proliferation, and features of cellular senescence. (a) Representative western blot showing phosphorylated AKT (p-AKT Ser473) and total AKT (t-AKT) in total lysates from control and CMT4B3 fibroblasts. Densitometric analysis of the p-AKT:T-AKT ratio was performed after normalization to GAPDH. Data is presented as mean ± SD from four independent experiments. (b) Growth curves and population doubling time (PDT) of control and CMT4B3 fibroblasts seeded at 6000 cells/well in 24-well plates and cultured for 3 days in complete DMEM with 10% FBS. PDT was calculated between day 1 and day 2 by direct cell counting using the following formula: PD = t × log2/(logC2 − logC1), where t = 24 h, and C1 and C2 are the cell counts at day 1 and day 2, respectively. Data are presented as mean ± SD from three independent experiments. (c) Representative images of senescence-associated β-galactosidase (SA-β-gal) staining in control and CMT4B3 fibroblasts. Quantification of SA-β-gal-positive cells is shown in the adjacent bar graph. Data are presented as mean ± SD from three independent experiments. (d) Representative western blot of p53 and p21 in total lysates from control and patient-derived fibroblasts. Densitometric quantification was normalized to GAPDH. Data is presented as mean ± SD from three independent experiments. Statistical significance was assessed using Student’s *t-*test (^*^P < .05; ^**^P < .01; ^***^P < .001; ^****^P < .0001).

Given the observed reduction in ATP production and the quiescent metabolic phenotype, we assessed functional consequences at the cellular level. Growth curves in glucose medium showed that CMT4B3 fibroblasts entered logarithmic growth later and proliferated more slowly than controls, with a 43% increase in population doubling time between days 1 and 3 post-seeding (Fig. 5b). Since prolonged doubling time and growth arrest characterize senescence, we evaluated senescence-associated β-galactosidase (SA-βGal) activity, finding 89.6% SA-βGal-positive cells in patient fibroblasts (Fig. 5c), indicative of premature senescence driven by metabolic dysfunction. Molecular analysis supported this, revealing significantly elevated p53 (+56.7%) and p21^WAF1/CIP1 (+49%) protein levels in CMT4B3 *versus* control cells (Fig. 5d). Collectively, these data demonstrate that chronic mitochondrial impairment in MTMR5/SBF1-mutated fibroblasts delays proliferation and activates premature senescence through the p53/p21^WAF1/CIP1 pathway.

## Discussion

Biallelic mutations in *MTMR5/SBF1* cause CMT4B3, a very rare subtype of Charcot–Marie–Tooth disease with highly variable clinical features, ranging from early-onset neurodevelopmental manifestations to late-onset peripheral neuropathy. Although initially classified as a demyelinating neuropathy, multiple reports, including the present case originally described by Berti et al. [23], have documented mixed or predominantly axonal features, supporting a broader and more heterogeneous disease spectrum [2, 23, 24]. While a loss-of-function mechanism has been proposed, the cellular pathways underlying disease pathogenesis remain poorly understood.

In this study, we performed functional, transcriptomic, and molecular analyses in fibroblasts derived from a CMT4B3 patient carrying *MTMR5/SBF1* mutations to investigate disease-associated cellular phenotypes. Our findings link mitochondrial fragmentation, defective metabolic adaptation, and altered cellular signalling in CMT4B3 fibroblasts. Patient-derived cells showed a coordinated phenotype characterized by fission-driven mitochondrial fragmentation, impaired glycolytic compensation associated with energetic failure, and dysregulated PI3K/AKT signalling, together with the emergence of a senescence-like state, highlighting previously unrecognized aspects of CMT4B3 cellular dysfunction. Patient-derived fibroblasts showed reduced proliferation and morphological abnormalities, together with molecular markers of senescence, including increased SA-β-galactosidase activity and elevated p53 and p21 expression. These alterations occurred in the absence of overt oxidative stress or DNA damage, supporting the presence of a metabolically driven stress-associated senescence phenotype. Mitochondrial dysfunction emerged as a prominent feature, with CMT4B3 fibroblasts displaying mitochondrial fragmentation and markedly impaired OxPhos, resulting in significant ATP depletion despite preserved respiratory chain complex assembly.

This apparent discrepancy suggests that the bioenergetic impairment observed in CMT4B3 fibroblasts is unlikely to arise from defective respiratory complex biogenesis or assembly. Instead, it may reflect reduced functional efficiency associated with mitochondrial network fragmentation and altered organelle architecture. Mitochondrial fragmentation may compromise the spatial organization of the inner mitochondrial membrane, potentially affecting oxidative phosphorylation efficiency despite structurally intact respiratory complexes. Although the precise mechanisms remain to be established, these observations suggest that alterations in mitochondrial architecture may contribute to the impaired bioenergetic phenotype. MTMR5/SBF1, in association with MTMR2, regulates local phosphoinositide pools at endosomal membranes, and dysregulation of these lipids may influence ER–mitochondria contacts, thereby affecting mitochondrial dynamics and metabolism without directly altering respiratory chain assembly. Although speculative, this interpretation is consistent with growing evidence indicating that endosomal lipid signalling contributes to the regulation of organelle interactions and metabolic adaptation [38, 39]. Consistent with patient-derived muscle and fibroblast data previously reported by Berti et al. [23] and Komilova et al. [28], our findings further support mitochondrial dysfunction as a central component of CMT4B3 pathogenesis.

Mitochondrial alterations are also widely implicated in several neurological disorders, including multiple CMT subtypes [32, 40, 41]. Despite the energy deficit, mitochondrial membrane potential (ΔΨm) remained preserved, and ROS levels were not increased, suggesting the activation of compensatory mechanisms, including reduced proton leak or increased resistance of the inner mitochondrial membrane, aimed at maintaining ΔΨm [42]. The distinct ROS/superoxide response following pyocyanin stimulation may indicate altered antioxidant buffering capacity. Overall, our findings support previous evidence suggesting that oxidative stress is not a central feature of CMT4B3 pathology [28]. Nevertheless, these compensatory responses appeared insufficient to restore energy homeostasis, resulting in persistent metabolic stress. Transcriptomic profiling supported this conclusion, revealing upregulation of *REDD1 (DDIT4)* and *EIF4EBP1*, canonical targets of energy stress associated with mTORC1 inhibition, together with downregulation of *PIK3R1* and *PIK3R3*, key regulatory subunits of the PI3K pathway. These changes pointed to disruption of the PI3K/AKT axis. Preferential phosphorylation of AKT at Ser473, an mTORC2-associated site, in the absence of activation at Thr308, the PDK1-dependent site, indicated incomplete AKT activation. The downregulation of ribosomal proteins such as *RPS4Y1* and *RPS9*, identified by transcriptomic analysis, further supports the presence of an energy-restricted cellular state. This altered signalling may contribute to impaired metabolic adaptation and to the senescence phenotype observed in CMT4B3 fibroblasts [43]. Further studies will be important to define better the role of PI3K/AKT signalling in these cellular responses. Notably, metabolic quiescence and growth arrest may also represent adaptive responses to chronic energetic stress, aimed at reducing ATP consumption while preserving cell viability. Accordingly, the senescence-like phenotype observed in CMT4B3 fibroblasts may reflect, at least in part, an adaptive response to sustained bioenergetic insufficiency. Further studies will be required to better define the relationship between these adaptive responses and the development of the senescence phenotype. Consistent with the observed mitochondrial defects, CMT4B3 fibroblasts exhibited increased mitophagy, as demonstrated by enhanced co-localization of mitochondria with autophagosomes and lysosomes [27]. This selective removal of dysfunctional mitochondria likely represents a compensatory attempt to preserve mitochondrial quality control, even in the absence of ΔΨm depolarization [44–46]. This phenotype resembles, although through distinct mechanisms, that previously observed in fibroblasts harbouring *OPA1* mutations, a mitochondrial fusion and cristae-structuring gene. As previously reported [26], *OPA1* mutations induce mitochondrial fragmentation, metabolic impairment, and p53/p21-mediated senescence associated with reduced AKT Ser473 phosphorylation. In contrast, *MTMR5/SBF1*-mutant fibroblasts displayed increased AKT Ser473 phosphorylation, suggesting dysregulated AKT-related signalling within an energy-stressed cellular context. These findings suggest that mitochondrial stress may differentially affect cellular signalling pathways depending on the underlying genetic defect. In *OPA1* mutant cells, the senescent phenotype appears associated with impaired adaptive signalling, whereas in *MTMR5/SBF1*-mutant fibroblasts, mitochondrial stress may still activate compensatory AKT-related pathways, although insufficiently to restore metabolic balance. A different cellular response has been described in *MFN2*-related neuropathy (CMT2A), where mitochondrial dysfunction promotes cellular proliferation rather than senescence, consistent with the proposed tumour suppressor role of MFN2 [25, 47]. Together, these observations support the concept that mitochondrial dysfunction can differentially modulate stress-response and metabolic signalling pathways involved in cellular adaptation and survival, depending on the specific molecular context [32, 33].

This study was performed using fibroblasts derived from a single CMT4B3 patient, reflecting the extreme rarity of the disease and the limited availability of biological material. This represents a limitation of the present work. While the observed cellular phenotype is consistent with the known pathogenic role of MTMR5/SBF1 deficiency, confirmation in additional patient-derived cell lines and genetic rescue experiments will be important to strengthen the causal relationship between MTMR5/SBF1 deficiency and the specific mitochondrial and senescence-associated alterations described here. Nevertheless, similar patient-derived fibroblast models have previously proven informative for investigating mitochondrial dysfunction and stress-response pathways in inherited neuropathies and mitochondrial disorders [25–27]. Additionally, recent studies have described mitochondrial alterations in fibroblasts carrying different MTMR5/SBF1 pathogenic variants [27, 28], supporting a consistent association between MTMR5/SBF1 mutations and mitochondrial dysfunction. Further studies using additional patient-derived and disease-relevant cellular models, together with iPSC-derived neuronal and Schwann cell systems and CRISPR-based genetic rescue approaches, will help to validate and extend these findings.

Overall, our findings indicate that *MTMR5/SBF1*-mutant fibroblasts exhibit altered mitochondrial metabolism, dysregulated PI3K/AKT signalling, and activation of mitochondrial quality control pathways associated with growth suppression and a senescence-like phenotype. Further studies using induced pluripotent stem cell (iPSC)-derived neuronal and Schwann cell models, together with genetic rescue approaches, will help clarify how these alterations contribute to the tissue-specific manifestations of CMT4B3 [48]. In post-mitotic, energy-demanding cells, persistent metabolic stress and mitochondrial dysfunction may ultimately compromise cellular viability and contribute to disease progression.

## Materials and methods

### Cell culture and treatments

Control fibroblasts included skin-derived cells from healthy individuals with no clinical or biochemical evidence of mitochondrial disease (Controls 1 and 2), as well as commercially available Normal Human Dermal Fibroblasts (NHDF, Control 3) purchased from Lonza (Walkersville, MD, USA). The age of the patient and controls at the time of skin biopsy was comparable. Informed consent was obtained from all individuals before the procedure.

Fibroblasts were cultured in high-glucose DMEM (EuroClone, ECB7501LX10) supplemented with 10% fetal bovine serum (FBS; EuroClone, ECS5000L), 1% L-glutamine (EuroClone, ECB3000D), 1% penicillin/streptomycin (EuroClone, ECB3001D), and 50 μg/mL uridine (Sigma-Aldrich, U3003). Cells were maintained at 37°C in a humidified atmosphere with 5% CO₂. To minimize the potential contribution of replicative senescence, patient-derived and control fibroblasts analysed within the same experiment were matched at equivalent passage numbers within the 8–12 passage range.

For experiments, cells were seeded onto sterile plastic dishes, flasks, or glass coverslips and allowed to adhere for at least 24 h before treatment. Where indicated, cells were cultured for 72 h in glucose-free DMEM supplemented with 5.5 mM galactose (DMEM-galactose) to assess mitochondrial network dynamics. Mitochondrial depolarization was induced by treatment with 10 μM carbonyl cyanide p-(trifluoromethoxy) phenylhydrazone (CCCP; Sigma-Aldrich, C2759) for 4 h. To evaluate mitochondrial morphology, cells were treated with the Drp1 inhibitor MDIVI-1 (Sigma-Aldrich, M0199) at a final concentration of 50 μM for 4 h.

Cell viability was assessed by manual cell counting. Live cells were identified by exclusion of 0.4% Trypan Blue (Sigma-Aldrich, T8154) and counted using a haemocytometer. For Seahorse metabolic assays, Control 1 was used because it was established using the same biopsy collection and culture procedures as the patient’s fibroblasts.

### Mitochondrial membrane potential (ΔΨm) assessment

Mitochondrial membrane potential was measured using the potentiometric dye tetramethylrhodamine ethyl ester (TMRE; Sigma-Aldrich, 87 917). Cells were incubated with 250 nM TMRE for 20 min at 37°C. To confirm ΔΨm sensitivity, oligomycin (3 μM; Sigma-Aldrich, O4876) was used to induce mitochondrial hyperpolarization; vehicle (DMSO) served as a control. Fluorescence intensity was recorded immediately and 15 min after treatment using a Cytation 5 Cell Imaging Multi-Mode Reader (BioTek Instruments) with a 20× objective. Image acquisition and analysis were performed with the integrated Gen5 software.

Quantification of TMRE fluorescence was performed on at least 100 cells per condition across three independent experiments. Mitochondrial morphology was evaluated using the Mitochondrial Morphology macro in ImageJ, based on at least 20 different fluorescent images per experimental group. The proportion of cells displaying hyperfused mitochondrial networks was quantified at 20× magnification.

### Fluorescence detection of reactive oxygen species (ROS and Superoxide)

Intracellular ROS levels were measured using the ROS-ID® Total ROS/Superoxide Detection Kit (ENZ-51010, Enzo Life Sciences, NY, USA) according to the manufacturer’s instructions. Equal numbers of cells were seeded into 96-well black-wall/clear-bottom plates and cultured to reach ~ 70–80% confluency at the time of analysis. Pyocyanin (200 μM) was used to induce ROS production, while the controls received vehicles alone (anhydrous dimethylformamide, DMF). After 30 min incubation at 37°C with the detection solution, fluorescence signals corresponding to general ROS and superoxide production were acquired using a Cytation 5 Cell Imaging Multi-Mode Reader (BioTek Instruments, VT, USA). Data were normalized to cell number based on Hoechst 33342 staining (Thermo Fisher Scientific, #14533) to account for cell density variability.

### ATP quantification by luminescence

Cellular ATP content was quantified using the ATP Determination Kit (A22066, Molecular Probes, Thermo Fisher Scientific), following the manufacturer’s protocol. Fibroblasts (2000 cells/well) were seeded in 96-well plates and cultured for 48 h. Subsequently, cells were incubated for 2 h in buffer (156 mM NaCl, 3 mM KCl, 2 mM MgSO₄, 1.25 mM KH₂PO₄, 2 mM CaCl₂, 20 mM HEPES, pH 7.35) supplemented with one of the following conditions: (i) 10 mM glucose; (ii) 10 mM glucose +2.5 μg/mL oligomycin (to assess glycolytic ATP); (iii) 5 mM 2-deoxy-D-glucose +5 mM pyruvate (to assess oxidative ATP). After incubation, cells were lysed, and the lysates were centrifuged at 12000 × g for 5 min at 4°C. Supernatants were incubated with the luciferin/luciferase reagent, and luminescence was measured using the Cytation 5 Reader. ATP concentrations were determined by interpolation from an ATP standard curve and normalized to cell number using Hoechst 33342 staining.

### Blue native-PAGE (BN-PAGE)

BN-PAGE was performed on mitochondrial fractions to evaluate the assembly and abundance of oxidative phosphorylation complexes (RCs) and supercomplexes (SCs). Mitochondrial mitoplasts were isolated following digitonin (8 mg/mL) treatment to selectively permeabilize the outer mitochondrial membrane. The resulting mitoplasts were resuspended in solubilization buffer (1.5 M aminocaproic acid, 50 mM Bis-Tris, 20 μM PMSF, pH 7.0). Protein concentrations were measured using the Bradford assay, and solubilization was achieved using either n-dodecyl-β-D-maltoside (DDM) or digitonin, depending on the target complex. Samples (20–50 μg) were loaded on 3–13% (for SCs) or 4–13% (for RCs) homemade gradient polyacrylamide gels and run at 4–7°C. After initial migration at 90 V, the cathode buffer was replaced with one containing lower Serva Blue G (0.002%), and electrophoresis continued at 200 V for 3 h.

In-Gel Activity staining for the enzymatic activities of mitochondrial complexes was assessed directly on BN-PAGE gels. Complex I (NADH: ubiquinone oxidoreductase): gels were incubated in 5 mM Tris–HCl (pH 7.4) containing 0.14 mg/mL NADH and 1 mg/mL nitro blue tetrazolium (NBT). Enzymatic activity was visualized as a dark-blue precipitate. Complex IV (cytochrome c oxidase): gels were incubated overnight in 50 mM potassium phosphate buffer (pH 7.2) containing 1 mg/mL diaminobenzidine (DAB), 1 mg/mL cytochrome c, 24 U/mL catalase, and 75 mg/mL sucrose. Activity was visualized as a brown precipitate. Complex V (ATP synthase): gels were incubated in 35 mM Tris–HCl (pH 7.8), 270 mM glycine, 14 mM MgSO₄, 0.2% Pb(NO₃)₂, and 8 mM ATP. Active bands appeared light blue. Coomassie-stained gels were imaged using a Bio-Rad ChemiDoc system.

### mtDNA analysis by long-range and quantitative PCR

Total DNA was isolated from fibroblasts using the Wizard® Genomic DNA Purification Kit (Promega, A1125). DNA concentration and purity were assessed by NanoDrop (ND-1000, Thermo Fisher Scientific), and integrity was checked by 0.8% agarose gel electrophoresis.

Large-scale mtDNA deletions were screened using long-range PCR, and mtDNA copy number was quantified by real-time PCR, as previously described [25, 26].

### Mitochondrial bioenergetics via seahorse XF cell Mito stress test

Mitochondrial function was assessed using the Seahorse XF Cell Mito Stress Test (Agilent, 103 015–100). Fibroblasts (3 × 10^4^/well) were seeded in XF24 Cell Culture Microplates (Agilent, 102 340–100) and incubated overnight. One hour before measurement, the culture medium was replaced with Seahorse XF DMEM Assay Medium (pH 7.4) supplemented with 10 mM glucose, 1 mM sodium pyruvate, and 2 mM glutamine. OCR and ECAR were measured using an XF24 Analyzer (Seahorse Bioscience) under basal conditions and after sequential injections of oligomycin (1.5 μM), FCCP (2 μM), and rotenone/antimycin A (0.5 μM each). Data were normalised to cell number via Hoechst 33342 staining and analyzed with Seahorse Wave software.

### Real-time ATP rate assay

The Seahorse Real-Time ATP Rate Assay was used to dissect mitochondrial and glycolytic contributions to cellular ATP production. Cells were incubated in assay medium containing 25 mM glucose, 10 mM sodium pyruvate, and 2 mM glutamine (pH 7.4). Sequential injections of oligomycin (1.5 μM) and rotenone/antimycin A (0.5 μM) allowed real-time calculation of glycolytic ATP (glycoATP), mitochondrial ATP (mitoATP), and total ATP production rate. Data were normalized to Hoechst-stained nuclei and analyzed using Seahorse Wave software.

### Immunoblotting

Cells were harvested at confluence, washed twice in DPBS (Euroclone, ECB4004L), and lysed in RIPA buffer (Thermo Fisher Scientific, #89900) supplemented with protease and phosphatase inhibitors (Cell Signaling, #5871 and #5870S). Lysates were sonicated on ice, centrifuged at 16000 × g for 10 min at 4°C, and protein concentration was determined by Bradford assay (Bio-Rad, #500–0006). Twenty to fifty micrograms of total protein were denatured in Laemmli buffer (4×, Bio-Rad, #1610737), separated on SDS-PAGE gels (8–12%) and transferred to PVDF membranes using the Trans-Blot apparatus (Bio-Rad). Membranes were blocked in TTBS (Tris-buffered saline with 0.1% Tween-20) with 5% non-fat dry milk and incubated overnight at 4°C with primary antibodies (listed in Table S4) diluted in TTBS containing 2.5% milk. After washing, membranes were incubated with appropriate HRP-conjugated secondary antibodies and developed by chemiluminescence.

### Determination of medium lactate concentration

To measure lactate concentrations, 2000 cells were seeded in a 96-well plate in DMEM culture medium. Lactic acid quantification was performed using high-performance liquid chromatography (HPLC) on a Nexera Lite system (Shimadzu), equipped with a Rezex ROA-Organic Acid H^+^ (8%) column (300 mm × 7.8 mm, Phenomenex). A 10 μL sample volume was injected, with 2.5 mM H₂SO₄ as the mobile phase phase at a flow rate of 0.6 mL/min. The column temperature was maintained at 65°C, and detection was carried out at 210 nm using a photodiode array detector (Shimadzu SPD-M40). All analyses were conducted in triplicate, and quantification was based on the external standard method.

### Immunofluorescence assays

The primary antibody anti-VDAC1/Porin was used for the mitochondrial network morphology study (Table S4). After three washes in PBS, cells were reacted with secondary antibody conjugated with Alexa Fluor 488 (Thermo Fisher Scientific, A11001) for 1 hour at room temperature in a humid chamber. Coverslips were mounted in Vectashield (Vector, H-100-10). For mitochondrial network evaluation, images were acquired at 60× magnification (Nikon×60 Plan Apocr λ NA = 1.40 or Leica TCS SP8 oil immersion objective). Three different mitochondrial morphology groups were outlined: tubular mitochondria (circularity 0–0.3), intermediate mitochondria (circularity 0.3–0.6), and fragmented mitochondria (circularity 0.6–1) as described elsewhere [49]. Mitochondrial circularity was quantified by using ImageJ software (https://imagej.nih.gov/ij/). For each experiment, at least 20 cells were used to calculate the relative percentages of the mitochondrial areas for the different mitochondrial groups, normalised on the total area of the mitochondrial compartment.

### Transcriptome profiling and functional analysis of DEGs

RNA was extracted from patient-derived fibroblasts and control fibroblasts using the RNeasy Plus Kit (Qiagen, 74 034) according to the manufacturer’s instructions. Each RNA sample was qualitatively and quantitatively checked on an Agilent 2100 Bioanalyzer RNA Nano Chip (Agilent, Santa Clara, CA, USA). Directional RNA-Seq libraries were prepared from 100 ng of total RNA using the TruSeq Stranded Total RNA Sample Prep Kit (Illumina, 20 020 597) according to the manufacturer’s protocol. Sequencing was performed on an Illumina NextSeq 500 platform (Illumina, San Diego, CA). RNA-Seq reads in FASTQ format were initially inspected using the FASTQC program (http://www.bioinformatics.babraham.ac.uk/projects/fastqc/). Adaptors and low-quality regions (Phred cutoff of 20) were trimmed using fastp [50], excluding reads with a final length of fewer than 50 bases. Cleaned reads were aligned onto the complete human genome (assembly hg38) using STAR [51] and provided a list of exon-exon junctions from GENCODE. The generalized fold change algorithm (GFOLD) was used to assign reads to known human annotations and to perform differential gene expression analysis between patient-derived fibroblasts and control fibroblasts. Only genes with a GFOLD (adjusted log₂ fold change) value equal to or greater than 1.5 in absolute value were considered. Functional annotation of DEGs was conducted using the online software DAVID (https://david.ncifcrf.gov/tools.jsp, 2021 Update), and network analysis as well as protein–protein interaction (PPI) analysis among DEGs was performed as previously described [25, 26]. DEGs were uploaded into the IPA tool along with their FC for pathway analysis.

### Growth curves and cell doubling time

To calculate the doubling time, the cell counting method and the following formula were used: PD = t x Log2/(LogC2-LogC1) [PD = population doubling time, t = 24 hours (interval of cell count), Log = 10-based log, C1 = 1st cell count, C2 = 2nd cell count]. To calculate the doubling level (PDL), the equation: PDL = 3.32× logN/No (N = harvest cell number and No = initial cell number) was used [52]. To obtain an accurate measurement of the PDL, the number of cells attached to the dish after seeding, rather than the number of cells seeded, was counted [52]. Patient-derived fibroblasts and control cells were seeded in 24-well plates and grown for 3 or 5 days in normal culture conditions. PD time was calculated from day 1 to day 2 for CMT4B3 cells. Cells were examined with Zeiss Primovert (Zeiss, Germany). A total of three individual experiments were performed.

### Detection of senescence-associated β-galactosidase in fibroblasts

The cells were washed using PBS and then fixed for 3–5 minutes at room temperature using 3% formaldehyde. After washing, they were incubated at 37°C (without CO2) with a fresh solution of senescence-associated β-Gal (SA-β-Gal) stain. This solution consisted of 1 mg of 5-bromo-4-chloro-3-indolyl β-D-galactoside (X-Gal) per ml (stock = 50 mg of dimethylformamide per ml), 40 mM citric acid/sodium phosphate, pH 6.0, 5 mM potassium ferrocyanide, 5 mM potassium ferricyanide, 150 mM NaCl, and 2 mM MgCl2. After 24 hours, staining became evident, and only senescent cells showed blue signals that could be detected using bright-field microscopy. To determine the percentage of SA-β-Gal-positive cells, the cells in 3 random fields (at least 100 cells) were counted using a Carl Zeiss Primovert microscope (10×).

### Statistical analysis

All statistical analyses were performed using PRISM® 7.04 in analytical software (GraphPad Software Inc., San Diego, CA) and Excel (Microsoft, Inc.). Results were expressed as average values ± SD of at least three independent determinations, each performed in triplicate, if not otherwise specified, using sex and age-matched control and patient-derived fibroblasts. Normality was assessed using the Shapiro–Wilk test. Normally distributed data were analyzed using Student’s *t-*test, whereas non-Gaussian data were analyzed using the Mann–Whitney non-parametric test. Statistical significance was set at: ^*^P < .05; ^**^P < .01; and ^***^P < .001; and ^****^P < .0001. A one-way analysis of variance (ANOVA) test was performed to examine the differences between more than two dependent groups. The Bonferroni correction was used for multiple comparisons.

## Supplementary Material

Supplementary_File_S2_HMG_submitted_clean_ddag080

## Data Availability

The data generated and analysed during this study are included in this published article and its supplementary materials. Additional datasets are available from the corresponding author upon reasonable request.

## References

[1] Pareyson D, Scaioli V, Laurà M. Clinical and electrophysiological aspects of Charcot-Marie-tooth disease. NeuroMolecular Med 2006;8:3–22. 10.1385/NMM:8:1-2:3. 16775364

[2] Pareyson D, Stojkovic T, Reilly MM. et al. A multicenter retrospective study of charcot-marie-tooth disease type 4B (CMT4B) associated with mutations in myotubularin-related proteins (MTMRs). Ann Neurol 2019;86:55–67. 10.1002/ana.25500. 31070812 PMC6581441

[3] Bertini A, Reilly MM, Pisciotta C. et al. Disease progression in Charcot-Marie-tooth disease type 4B (CMT4B) associated with mutations in myotubularin-related proteins 2 and 13. Eur J Neurol 2025;32:e70084. 10.1111/ene.70084. 39943887 PMC11822262

[4] Vergne I, Deretic V. The role of PI3P phosphatases in the regulation of autophagy. FEBS Lett 2010;584:1313–1318. 10.1016/j.febslet.2010.02.054. 20188094 PMC2885894

[5] Blondeau F, Laporte J, Bodin S. et al. Myotubularin, a phosphatase deficient in myotubular myopathy, acts on phosphatidylinositol 3-kinase and phosphatidylinositol 3-phosphate pathway. Hum Mol Genet 2000;9:2223–2229. 10.1093/oxfordjournals.hmg.a018913. 11001925

[6] Robinson FL, Dixon JE. Myotubularin phosphatases: policing 3-phosphoinositides. Trends Cell Biol 2006;16:403–412. 10.1016/j.tcb.2006.06.001. 16828287

[7] Clague MJ, Lorenzo Ó. The myotubularin family of lipid phosphatases. Traffic 2005;6:1063–1069. 10.1111/j.1600-0854.2005.00338.x. 16262718

[8] Wang J, Guo W, Wang Q. et al. Recent advances of myotubularin-related (MTMR) protein family in cardiovascular diseases. Front Cardiovasc Med 2024;11:1364604. 10.3389/fcvm.2024.1364604. 38529329 PMC10961392

[9] Hirsch E, Gulluni F, Martini M. Phosphoinositides in cell proliferation and metabolism. Adv Biol Regul 2020;75:100693. 10.1016/j.jbior.2020.100693. 32008962

[10] Posor Y, Jang W, Haucke V. Phosphoinositides as membrane organizers. Nat Rev Mol Cell Biol 2022;23:797–816. 10.1038/s41580-022-00490-x. 35589852 PMC9117997

[11] Palamiuc L, Ravi A, Emerling BM. Phosphoinositides in autophagy: current roles and future insights. FEBS J 2020;287:222–238. 10.1111/febs.15127. 31693781 PMC9154050

[12] Hou X, Chen Y, Carrillo ND. et al. Phosphoinositide signaling at the cytoskeleton in the regulation of cell dynamics. Cell Death Dis 2025;16:296. 10.1038/s41419-025-07616-x. 40229242 PMC11997203

[13] Toker A . Phosphoinositides and signal transduction. Cell Mol Life Sci 2002;59:761–779. 10.1007/s00018-002-8465-z. 12088277 PMC11146133

[14] Nandurkar HH, Layton M, Laporte J. et al. Identification of myotubularin as the lipid phosphatase catalytic subunit associated with the 3-phosphatase adapter protein, 3-PAP. Proc Natl Acad Sci 2003;100:8660–8665. 10.1073/pnas.1033097100. 12847286 PMC166368

[15] Kim S-A, Vacratsis PO, Firestein R. et al. Regulation of myotubularin-related (MTMR)2 phosphatidylinositol phosphatase by MTMR5, a catalytically inactive phosphatase. Proc Natl Acad Sci 2003;100:4492–4497. 10.1073/pnas.0431052100. 12668758 PMC153583

[16] Mammel AE, Delgado KC, Chin AL. et al. Distinct roles for the Charcot–Marie–tooth disease-causing endosomal regulators Mtmr5 and Mtmr13 in axon radial sorting and Schwann cell myelination. Hum Mol Genet 2022;31:1216–1229. 10.1093/hmg/ddab311. 34718573 PMC9190308

[17] Nakhro K, Park J-M, Hong YB. et al. SET binding factor 1 (*SBF1*) mutation causes Charcot-Marie-tooth disease type 4B3. Neurology 2013;81:165–173. 10.1212/WNL.0b013e31829a3421. 23749797

[18] Mégarbané A, Dorison N, Rodriguez D. et al. Multiple cranial nerve neuropathies, microcephaly, neurological degeneration, and “fork and bracket sign” in the MRI: a distinct syndrome. Am J Med Genet A 2010;152A:2297–2300. 10.1002/ajmg.a.33417. 20658556

[19] Bohlega S, Alazami A, Cupler E. et al. A novel syndromic form of sensory-motor polyneuropathy is linked to chromosome 22q13.31-q13.33. Clin Genet 2011;79:193–195. 10.1111/j.1399-0004.2010.01524.x. 21210780

[20] Alazami AM, Alzahrani F, Bohlega S. et al. SET binding factor 1 (*SBF1*) mutation causes Charcot-Marie-tooth disease type 4B3. Neurology 2014;82:1665–1666. 10.1212/WNL.0000000000000331. 24799518

[21] Manole A, Horga A, Gamez J. et al. SBF1 mutations associated with autosomal recessive axonal neuropathy with cranial nerve involvement. neurogenetics 2017;18:63–67. 10.1007/s10048-016-0505-1. 28005197

[22] Flusser H, Halperin D, Kadir R. et al. Novel *SBF1* splice-site null mutation broadens the clinical spectrum of Charcot-Marie-tooth type 4B3 disease. Clin Genet 2018;94:473–479. 10.1111/cge.13419. 30039846

[23] Berti B, Longo G, Mari F. et al. Bi-allelic variants in MTMR5/SBF1 cause Charcot-Marie-tooth type 4B3 featuring mitochondrial dysfunction. BMC Med Genet 2021;14:157. 10.1186/s12920-021-01001-1. PMC8199524 34118926

[24] Silva L, Igreja L, Cardoso M. et al. Charcot-Marie-tooth 4B3 phenotype expansion and confounding factors. Curr Genet Med Rep 2026;14:5. 10.1007/s40142-026-00236-7.

[25] Zanfardino P, Longo G, Amati A. et al. Mitofusin 2 mutation drives cell proliferation in Charcot-Marie-tooth 2A fibroblasts. Hum Mol Genet 2023;32:333–350. 10.1093/hmg/ddac201 35994048

[26] Zanfardino P, Amati A, Doccini S. et al. *OPA* 1 mutation affects autophagy and triggers senescence in autosomal dominant optic atrophy *plus* fibroblasts. Hum Mol Genet 2024;33:768–786. 10.1093/hmg/ddae008. 38280232

[27] Zanfardino P, Amati A, Doccini S. et al. Selective mitophagy activation and protein aggregate accumulation in MTMR5/SBF1-deficient fibroblasts. Life Sci 2025;381:123995. 10.1016/j.lfs.2025.123995. 40998285

[28] Komilova NR, Angelova PR, Cali E. et al. Charcot Marie tooth disease pathology is associated with mitochondrial dysfunction and lower glutathione production. Cell Mol Life Sci 2025;82:72. 10.1007/s00018-025-05612-0. 39918771 PMC11806186

[29] Bordt EA, Clerc P, Roelofs BA. et al. The putative Drp1 inhibitor mdivi-1 is a reversible mitochondrial complex I inhibitor that modulates reactive oxygen species. Dev Cell 2017;40:583–594.e6. 10.1016/j.devcel.2017.02.020. 28350990 PMC5398851

[30] Acín-Pérez R, Fernández-Silva P, Peleato ML. et al. Respiratory active mitochondrial Supercomplexes. Mol Cell 2008;32:529–539. 10.1016/j.molcel.2008.10.021. 19026783

[31] Calvaruso MA, Smeitink J, Nijtmans L. Electrophoresis techniques to investigate defects in oxidative phosphorylation. Methods 2008;46:281–287. 10.1016/j.ymeth.2008.09.023. 18948205

[32] Van Lent J, Verstraelen P, Asselbergh B. et al. Induced pluripotent stem cell-derived motor neurons of CMT type 2 patients reveal progressive mitochondrial dysfunction. Brain 2021;144:2471–2485. 10.1093/brain/awab226. 34128983 PMC8418338

[33] Kazyken D, Magnuson B, Bodur C. et al. AMPK directly activates mTORC2 to promote cell survival during acute energetic stress. Sci Signal 2019;12:eaav3249. 10.1126/scisignal.aav3249. 31186373 PMC6935248

[34] Burgering BMT, Coffer PJ. Protein kinase B (c-Akt) in phosphatidylinositol-3-OH kinase signal transduction. Nature 1995;376:599–602. 10.1038/376599a0. 7637810

[35] Sarbassov DD, Guertin DA, Ali SM. et al. Phosphorylation and regulation of Akt/PKB by the Rictor-mTOR complex. Science 2005;307:1098–1101. 10.1126/science.1106148. 15718470

[36] Sarbassov DD, Ali SM, Sengupta S. et al. Prolonged rapamycin treatment inhibits mTORC2 assembly and Akt/PKB. Mol Cell 2006;22:159–168. 10.1016/j.molcel.2006.03.029. 16603397

[37] Hresko RC, Mueckler M. mTOR·RICTOR is the Ser473 kinase for Akt/protein kinase B in 3T3-L1 adipocytes. J Biol Chem 2005;280:40406–40416. 10.1074/jbc.M508361200. 16221682

[38] Lourdes SR, Gurung R, Giri S. et al. A new role for phosphoinositides in regulating mitochondrial dynamics. Adv Biol Regul 2024;91:101001. 10.1016/j.jbior.2023.101001. 38057188

[39] Jang W, Puchkov D, Samsó P. et al. Endosomal lipid signaling reshapes the endoplasmic reticulum to control mitochondrial function. Science 2022;378:eabq5209. 10.1126/science.abq5209. 36520888

[40] Kalmar B, Innes A, Wanisch K. et al. Mitochondrial deficits and abnormal mitochondrial retrograde axonal transport play a role in the pathogenesis of mutant Hsp27-induced Charcot Marie tooth disease. Hum Mol Genet 2017;26:3313–3326. 10.1093/hmg/ddx216. 28595321 PMC5808738

[41] Tucci A, Liu Y-T, Preza E. et al. Novel C12orf65 mutations in patients with axonal neuropathy and optic atrophy. J Neurol Neurosurg Psychiatry 2014;85:486–492. 10.1136/jnnp-2013-306387. 24198383 PMC3995331

[42] Sakamuri SS, Sure VN, Kolli L. et al. Aging related impairment of brain microvascular bioenergetics involves oxidative phosphorylation and glycolytic pathways. J Cereb Blood Flow Metab 2022;42:1410–1424. 10.1177/0271678X211069266. 35296173 PMC9274865

[43] Yu L, Chen X, Sun X. et al. The glycolytic switch in tumors: how many players are involved? J Cancer 2017;8:3430–3440. 10.7150/jca.21125. 29151926 PMC5687156

[44] Trempe J-F, Gehring K. Structural mechanisms of mitochondrial quality control mediated by PINK1 and parkin. J Mol Biol 2023;435:168090. 10.1016/j.jmb.2023.168090. 37054910

[45] Barazzuol L, Giamogante F, Brini M. et al. PINK1/parkin mediated mitophagy, Ca2+ signalling, and ER–mitochondria contacts in Parkinson’s disease. Int J Mol Sci 2020;21:1772. 10.3390/ijms21051772. 32150829 PMC7084677

[46] Yu Z, Wang H, Tang W. et al. Mitochondrial Ca2+ oscillation induces mitophagy initiation through the PINK1-parkin pathway. Cell Death Dis 2021;12:632. 10.1038/s41419-021-03913-3. 34148057 PMC8214625

[47] Zanfardino P, Amati A, Perrone M. et al. The balance of MFN2 and OPA1 in mitochondrial dynamics, cellular homeostasis, and disease. Biomolecules 2025;15:433. 10.3390/biom15030433. 40149969 PMC11940761

[48] Maria Turco E, Maria Giada Giovenale A, Rotundo G. et al. Generation and characterization of CSSi016-a (9938) human pluripotent stem cell line carrying two biallelic variants in MTMR5/SBF1 gene resulting in a case of severe CMT4B3. Stem Cell Res 2022;65:102946. 10.1016/j.scr.2022.102946. 36272304

[49] Pezzini I, Marino A, Del Turco S. et al. Cerium oxide nanoparticles: the regenerative redox machine in bioenergetic imbalance. Nanomed. 2017;12:403–416. 10.2217/nnm-2016-0342. 28000542

[50] Chen S, Zhou Y, Chen Y. et al. Fastp: an ultra-fast all-in-one FASTQ preprocessor. Bioinformatics 2018;34:i884–i890. 10.1093/bioinformatics/bty560. 30423086 PMC6129281

[51] Dobin A, Davis CA, Schlesinger F. et al. STAR: ultrafast universal RNA-seq aligner. Bioinformatics 2013;29:15–21. 10.1093/bioinformatics/bts635. 23104886 PMC3530905

[52] Giovannelli L, Pitozzi V, Jacomelli M. et al. Protective effects of resveratrol against senescence-associated changes in cultured human fibroblasts. J Gerontol Ser A 2011;66A:9–18. 10.1093/gerona/glq161. 20884849

