# Supplementary material for: Mitochondrial dysfunction, metabolic quiescence and premature senescence in CMT4B3 fibroblasts
Source: Hum Mol Genet. 2026 Aug 21;35(17):ddag080. doi: 10.1093/hmg/ddag080 (PMC13648011; doi:10.1093/hmg/ddag080)
Supplement: Supplementary file 1 [file supplementary_file_s2_hmg_submitted_clean_ddag080.docx]

**Defective mitochondrial respiration and energy failure in *MTMR5/SBF1*-mutant fibroblasts associated with CMT4B3**

Paola Zanfardino^1^, Alessandro Amati^1^, Sharon Cox^2^, Gennaro Agrimi^2^, Stefano Doccini^3^, Patricio Fernández-Silva^4^, Apollonia Tullo^5^, Jessica Rosati^6^, Filippo M. Santorelli^3^, Vittoria Petruzzella^1^

*^1^ Department of Translational Biomedicine and Neuroscience (DiBraiN), University of Bari Aldo Moro, Bari, Italy*

*^2^Department of Biosciences, Biotechnology and Environment, University of Bari Aldo Moro, 70125 Bari, Italy*

*^3^Molecular Medicine for Neurodegenerative and Neuromuscular Diseases Unit, IRCCS Stella Maris Foundation, Pisa, Italy*

*^4^Department of Biochemistry, University of Zaragoza; Institute for Biocomputation and Physics of Complex Systems, University of Zaragoza, Zaragoza, Spain*

*^5^Institute of Biomembranes, Bioenergetics and Molecular Biotechnologies, National Research Council, Bari, Italy*

*^6^Cellular Reprogramming Unit, Fondazione IRCCS Casa Sollievo della Sofferenza, San Giovanni Rotondo, Italy*

**Figure S1. Lactate production is reduced in CMT4B3 fibroblasts.** Time-course traces for the lactate production at 7, 24, 48 and 72 hours in controls and CMT4B3 fibroblasts.

**Figure S2. ROS and superoxide production are not altered in CMT4B3 fibroblasts**

Bar graphs quantifying reactive oxygen species (ROS) and superoxide levels in control and CMT4B3 fibroblasts, measured by fluorescence using a microplate reader under basal conditions (*Untr*) and after treatment with pyocyanin (*PYO*).

Data is presented as mean ± SD from at least three independent experiments (n ≥ 3). Statistical significance was assessed using Student’s t-test (*p < 0.05; **p < 0.01; ***p < 0.001; ****p < 0.0001). For multiple comparisons, one-way ANOVA with Bonferroni’s post hoc test was applied.

**Figure S3. Mitochondrial complex expression, mtDNA integrity, and copy number are preserved in CMT4B3 fibroblasts**

(**a**) Representative BN-PAGE showing oxidative phosphorylation (OXPHOS) complexes and supercomplexes in mitochondrial mitoplasts from control and CMT4B3 fibroblasts. Mitoplasts were solubilized with digitonin (DIG 1%) to preserve and analyse Complex I-containing supercomplexes or with n-dodecyl-β-D-maltoside (DDM 1%) to resolve individual respiratory chain complexes. From top to bottom: in-gel activity assay of Complex I-containing supercomplexes (DIG 1%), followed by in-gel activity assays of Complex I (CI), Complex IV (CIV), and Complex V (CV) (DDM 1%), each followed by Coomassie staining as a loading control.

(**b**) Representative Western blot of mitochondrial proteins in total cell lysates from control and patient-derived fibroblasts, including TOMM20 and subunits of CI (ND4, NDUFS1), CIV (COX4), and CV (ATP5F1B). Densitometric quantification was normalized to GAPDH signal and is shown in the accompanying bar graphs. Data are presented as mean ± SD from three (TOMM20, ND4, ATP5F1B), four (COX4), or six (NDUFS1) independent experiments.

(**c**) Long-range PCR for mtDNA integrity, showing a 16.5 kb amplicon corresponding to the full-length mitochondrial genome in control and CMT4B3 cells, visualized on a 0.8% agarose gel.

(**d**) Quantification of mtDNA copy number by qRT-PCR, expressed as the ratio of mitochondrial DNA to nuclear DNA (mtDNA:nDNA), indicative of the relative number of mtDNA copies per cell.

(**e**) Representative Western blot showing the expression of TFEB and mtTFA in total lysates from control and CMT4B3 fibroblasts. Densitometric analysis was normalized to GAPDH signal. Data are presented as mean ± SD from four (TFEB) or eight (mtTFA) independent experiments. Statistical significance was assessed using Student’s t-test (*p < 0.05; **p < 0.01; ***p < 0.001; ****p < 0.0001).

**Figure S4. Different pathways are impaired in CMT4B3 fibroblasts.** Representative images of axon guidance (**A**), cytokine-cytokine receptor interaction (**B**) and PI3K-AKT signaling (**C**) pathways from KEGG in CMT4B3 fibroblasts. Differentially expressed genes have a fold change > 1.5. Green genes are downregulated and red genes are upregulated.

**A**

**B**

**C**

**Figure S5. Schematic representation of the PI3K-AKT-mTOR pathway highlighting genes identified as differentially expressed through transcriptomic analysis.** The pathway is graphically represented as nodes (genes) and edges (the biological relationship between genes). Red and green shaded nodes represent upregulated and downregulated genes, respectively.

**Figure S6. Protein-protein interaction (PPI) network analysis shows the connectivity between differentially expressed genes in CMT4B3 fibroblasts.** The nodes and edges were constructed algorithmically using the g STRING tool and plotted using Cytoscape software. Red nodes represent upregulated genes, while blue nodes represent downregulated genes. Edges are shown in grey colour. The network contains six important gene clusters, which have been circled in pink and numbered. These clusters have an MCODE score greater than 5.

**Figure S7. Deep sequencing and in silico analyses of the compound heterozygous c.2291G>A/p.R763H and c.3194G>A/p.G1064E mutations of MTMR5/SBF1 in CMT4B3 fibroblasts.** RNA-seq reads from CMT4B3 fibroblasts were used to assess the ratio of mutant mRNA to wt mRNA. For the g.50460364C>T (GRCh38) (c.3194G>A) mutation, out of 149 total reads, 71 (48%) had the mutated variant T and 78 (52%) had the wild-type nucleotide. For the g.50462313C>T (GRCh38) (c.2288G>A) mutation, out of 192 total reads, 91 (47%) had the mutated variant T, 1 (1%) had the variant G and 100 (52%) had the wildtype nucleotide. No compound heterozygous mutations were found in control fibroblasts.

**Table S1** Functional and pathway enrichment analysis of differentially expressed genes (DEG) from Gene Ontology (GO) in CMT4B3 fibroblasts. DEGs were functionally annotated using the online software called DAVID (2021 Update) which extracts biological information from multiple genes. The software performs GO enrichment analysis for three aspects of biology: biological processes (BP), cellular components (CC), and molecular functions (MF). The software applies a Benjamini-Hochberg False Discovery Rate (FDR) of P<0.001 for selecting functional and pathway enrichment analyses.

| **Gene set name** | **Term** | | | **Gene Count** | | **%** | | **P*** | |  |
| --- | --- | --- | --- | --- | --- | --- | --- | --- | --- | --- |
| GO_TERM: BIOLOGICAL_PROCESS | | cell adhesion | | | 53 | | 6.0 | | 7.20E-06 | |
|  |  | extracellular matrix organization | | | 25 | | 2.8 | | 2.80E-05 | |
|  |  | signal transduction | | | 87 | | 9.8 | | 1.00E-04 | |
|  |  | BMP signaling pathway | | | 16 | | 1.8 | | 6.20E-04 | |
|  |  | positive regulation of gene expression | | | 44 | | 4.9 | | 6.20E-04 | |
|  |  | positive regulation of cell proliferation | | | 46 | | 5.2 | | 6.20E-04 | |
|  |  | brain development | | | 29 | | 3.3 | | 7.60E-04 | |
|  |  | angiogenesis | | | 27 | | 3.0 | | 1.70E-03 | |
|  |  | animal organ morphogenesis | | | 19 | | 2.1 | | 1.90E-03 | |
|  |  | collagen fibril organization | | | 12 | | 1.3 | | 4.80E-03 | |
|  |  | positive regulation of cell migration | | | 26 | | 2.9 | | 5.00E-03 | |
|  |  | positive regulation of cardiac muscle cell proliferation | | | 9 | | 1.0 | | 7.20E-03 | |
|  |  | secondary heart field specification | | | 6 | | 0.7 | | 8.10E-03 | |
|  |  | cell-cell adhesion | | | 21 | | 2.4 | | 9.40E-03 | |
|  |  | inner ear morphogenesis | | | 11 | | 1.2 | | 1.00E-02 | |
|  |  | response to glucocorticoid | | | 12 | | 1.3 | | 1.00E-02 | |
|  |  | cell fate commitment | | | 11 | | 1.2 | | 1.00E-02 | |
|  |  | positive regulation of angiogenesis | | | 19 | | 2.1 | | 1.00E-02 | |
|  |  | central nervous system development | | | 18 | | 2.0 | | 1.00E-02 | |
|  |  | positive regulation of Wnt signaling pathway | | | 9 | | 1.0 | | 1.60E-02 | |
|  |  | protein homooligomerization | | | 16 | | 1.8 | | 1.60E-02 | |
|  |  | cellular response to hormone stimulus | | | 8 | | 0.9 | | 1.70E-02 | |
|  |  | cardiac muscle cell proliferation | | | 7 | | 0.8 | | 1.90E-02 | |
|  |  | stem cell proliferation | | | 10 | | 1.1 | | 1.90E-02 | |
|  |  | pulmonary valve morphogenesis | | | 6 | | 0.7 | | 2.40E-02 | |
|  |  | sensory perception of pain | | | 10 | | 1.1 | | 2.80E-02 | |
|  |  | odontogenesis | | | 8 | | 0.9 | | 3.20E-02 | |
|  |  | tissue development | | | 8 | | 0.9 | | 3.70E-02 | |
|  |  | epithelial to mesenchymal transition | | | 9 | | 1.0 | | 3.70E-02 | |
|  |  | glial cell migration | | | 5 | | 0.6 | | 4.20E-02 | |
|  |  | response to estrogen | | | 11 | | 1.2 | | 4.20E-02 | |
|  |  | chondrocyte development | | | 6 | | 0.7 | | 4.90E-02 | |
| GO TERM: CELLULAR COMPONENT | | | extracellular region | | 166 | | 18.7 | | 7.40E-18 | |
|  |  |  | extracellular matrix | | 46 | | 5.2 | | 1.10E-15 | |
|  |  |  | extracellular space | | 146 | | 16.4 | | 3.60E-14 | |
|  |  |  | cell surface | | 66 | | 7.4 | | 2.90E-11 | |
|  |  |  | plasma membrane | | 279 | | 31.4 | | 3.20E-11 | |
|  |  |  | integral component of membrane | | 282 | | 31.7 | | 1.20E-08 | |
|  |  |  | integral component of plasma membrane | | 106 | | 11.9 | | 2.90E-08 | |
|  |  |  | glutamatergic synapse | | 36 | | 4 | | 3.90E-05 | |
|  |  |  | endoplasmic reticulum lumen | | 30 | | 3.4 | | 2.60E-04 | |
|  |  |  | anchoring junction | | 39 | | 4.4 | | 6.50E-04 | |
|  |  |  | receptor complex | | 23 | | 2.6 | | 1.00E-03 | |
|  |  |  | basement membrane | | 14 | | 1.6 | | 1.90E-03 | |
|  |  |  | Z disc | | 16 | | 1.8 | | 5.70E-03 | |
|  |  |  | endoplasmic reticulum | | 68 | | 7.6 | | 6.20E-03 | |
|  |  |  | postsynaptic density | | 24 | | 2.7 | | 6.20E-03 | |
|  |  |  | voltage-gated potassium channel complex | | 12 | | 1.3 | | 7.10E-03 | |
|  |  |  | sarcoplasmic reticulum | | 9 | | 1 | | 9.20E-03 | |
|  |  |  | postsynaptic membrane | | 16 | | 1.8 | | 2.40E-02 | |
|  |  |  | dendritic spine | | 16 | | 1.8 | | 4.30E-02 | |
|  |  |  | GABA-ergic synapse | | 10 | | 1.1 | | 4.30E-02 | |
|  |  |  | perikaryon | | 16 | | 1.8 | | 4.30E-02 | |
|  |  |  | sarcolemma | | 12 | | 1.3 | | 4.30E-02 | |
|  |  |  | intercalated disc | | 8 | | 0.9 | | 4.30E-02 | |
|  |  |  | caveola | | 10 | | 1.1 | | 4.30E-02 | |
|  |  |  | collagen trimer | | 11 | | 1.2 | | 4.30E-02 | |
|  |  |  | cytoskeleton | | 35 | | 3.9 | | 4.30E-02 | |
|  |  |  | axon | | 26 | | 2.9 | | 4.30E-02 | |
|  |  |  | adherens junction | | 16 | | 1.8 | | 4.30E-02 | |
|  |  |  | focal adhesion | | 29 | | 3.3 | | 4.60E-02 | |
| GO TERM : MOLECULAR FUNCTION | |  | | |  | |  | |  | |
|  |  | extracellular matrix structural constituent | | | 25 | | 2.8 | | 7.90E-07 | |
|  |  | heparin binding | | | 25 | | 2.8 | | 4.90E-05 | |
|  |  | growth factor activity | | | 24 | | 2.7 | | 4.90E-05 | |
|  |  | extracellular matrix binding | | | 10 | | 1.1 | | 4.00E-04 | |
|  |  | cytokine activity | | | 24 | | 2.7 | | 4.00E-04 | |
|  |  | receptor binding | | | 36 | | 4 | | 1.00E-03 | |
|  |  | calcium ion binding | | | 56 | | 6.3 | | 1.00E-03 | |
|  |  | integrin binding | | | 20 | | 2.2 | | 2.30E-03 | |
|  |  | metalloendopeptidase activity | | | 16 | | 1.8 | | 7.90E-03 | |
|  |  | aldehyde dehydrogenase (NAD) activity | | | 6 | | 0.7 | | 1.80E-02 | |
|  |  | voltage-gated potassium channel activity | | | 11 | | 1.2 | | 1.80E-02 | |
|  |  | delayed rectifier potassium channel activity | | | 8 | | 0.9 | | 2.00E-02 | |
|  |  | extracellular matrix structural constituent conferring compression resistance | | | 6 | | 0.7 | | 2.10E-02 | |
|  |  | protease binding | | | 14 | | 1.6 | | 2.40E-02 | |
|  |  | cell adhesion molecule binding | | | 11 | | 1.2 | | 2.50E-02 | |
|  |  | metallopeptidase activity | | | 11 | | 1.2 | | 2.60E-02 | |
|  |  | oxidoreductase activity. acting on the aldehyde or oxo group of donors. NAD or NADP as acceptor | | | 6 | | 0.7 | | 3.90E-02 | |
| KEGG PATHWAY | | Axon guidance | | | 26 | | 2.9 | | 1.00E-04 | |
|  |  | Cytokine-cytokine receptor interaction | | | 32 | | 3.6 | | 7.90E-04 | |
|  |  | PI3K-Akt signaling pathway | | | 33 | | 3.7 | | 7.80E-03 | |

* Benjamini-Hochberg False Discovery Rate (FDR) P<0.05

**Table S2. Functional and pathway enrichment analysis of differentially expressed genes (DEG) from Kyoto Encyclopedia of Genes and Genomes (KEGG) in CMT4B3 fibroblasts.** DEGs were functionally annotated using the online software called KEGG which identified three significantly impaired pathways: axon guidance, cytokine-cytokine receptor interaction and PI3K-AKT signaling pathway.

| **KEGG PATHWAY** | **OFFICIAL GENE SYMBOL** | **Gene Name** | **GFOLD(0.01)** |
| --- | --- | --- | --- |
| **Axon guidance** | ABLIM1 | actin binding LIM protein 1(ABLIM1) | -1.59514 |
|  | ABLIM2 | actin binding LIM protein family member 2(ABLIM2) | -1.56328 |
|  | EFNA5 | EPH receptor B6(EPHB6) | -1.57785 |
|  | EFNB2 | ephrin A5(EFNA5) | -2.90931 |
|  | EPHB6 | ephrin B2(EFNB2) | -3.75355 |
|  | MYL9 | myosin light chain 9(MYL9) | -1.50962 |
|  | NGEF | netrin 1(NTN1) | -5.37333 |
|  | NTN1 | netrin 4(NTN4) | -2.93712 |
|  | NTN4 | neuronal guanine nucleotide exchange factor(NGEF) | -1.69347 |
|  | PIK3R1 | patched 1(PTCH1) | -1.52064 |
|  | PIK3R3 | phosphoinositide-3-kinase regulatory subunit 1(PIK3R1) | -1.79428 |
|  | PLXNB1 | phosphoinositide-3-kinase regulatory subunit 3(PIK3R3) | -1.754 |
|  | PLXNB3 | plexin B1(PLXNB1) | -1.92423 |
|  | PLXNC1 | plexin B3(PLXNB3) | -2.43759 |
|  | PTCH1 | plexin C1(PLXNC1) | 1.55998 |
|  | RAC2 | Rac family small GTPase 2(RAC2) | 1.92819 |
|  | RGMA | repulsive guidance molecule BMP co-receptor a(RGMA) | -1.59274 |
|  | ROBO2 | roundabout guidance receptor 2(ROBO2) | -6.24299 |
|  | SEMA3B | semaphorin 3B(SEMA3B) | -2.0122 |
|  | SEMA3F | semaphorin 3F(SEMA3F) | -4.24596 |
|  | SEMA3G | semaphorin 3G(SEMA3G) | -3.74073 |
|  | SLIT2 | slit guidance ligand 2(SLIT2) | 3.82531 |
|  | SRGAP3 | SLIT-ROBO Rho GTPase activating protein 3(SRGAP3) | -2.03423 |
|  | TRPC4 | transient receptor potential cation channel subfamily C member 4(TRPC4) | 1.51648 |
|  | UNC5B | unc-5 netrin receptor B(UNC5B) | 2.52763 |
|  | WNT5A | Wnt family member 5A(WNT5A) | 1.92009 |
| Cytokine-cytokine receptor interaction | ACKR3 | atypical chemokine receptor 3(ACKR3) | -1.54764 |
|  | ACKR4 | atypical chemokine receptor 4(ACKR4) | 2.23216 |
|  | ACVR1C | activin A receptor type 1C(ACVR1C) | -2.00035 |
|  | BMP2 | bone morphogenetic protein 2(BMP2) | -2.14599 |
|  | BMP4 | bone morphogenetic protein 4(BMP4) | -2.69284 |
|  | CD70 | CD70 molecule(CD70) | -2.21101 |
|  | CSF2RB | colony stimulating factor 2 receptor subunit beta(CSF2RB) | 3.08637 |
|  | GDF1 | growth differentiation factor 1(GDF1) | -1.81501 |
|  | GDF6 | growth differentiation factor 6(GDF6) | 1.51586 |
|  | GHR | growth hormone receptor(GHR) | -2.98434 |
|  | IL11 | interleukin 11(IL11) | 1.97036 |
|  | IL1RAP | interleukin 1 receptor accessory protein(IL1RAP) | 1.79817 |
|  | IL20RB | interleukin 20 receptor subunit beta(IL20RB) | 1.81499 |
|  | IL27RA | interleukin 27 receptor subunit alpha(IL27RA) | 1.72542 |
|  | IL32 | interleukin 32(IL32) | 3.25364 |
|  | IL6 | interleukin 6(IL6) | 5.74887 |
|  | IL7 | interleukin 7(IL7) | -2.5209 |
|  | IL7R | interleukin 7 receptor(IL7R) | 2.14094 |
|  | INHBE | inhibin subunit beta E(INHBE) | 2.32758 |
|  | LEP | leptin(LEP) | -1.95427 |
|  | LIF | LIF interleukin 6 family cytokine(LIF) | 2.11278 |
|  | MSTN | myostatin(MSTN) | 1.84566 |
|  | PRLR | prolactin receptor(PRLR) | -2.16611 |
|  | TGFB2 | transforming growth factor beta 2(TGFB2) | -2.7137 |
|  | TNFRSF10A | TNF receptor superfamily member 10a(TNFRSF10A) | -1.53621 |
|  | TNFRSF11B | TNF receptor superfamily member 11b(TNFRSF11B) | 1.80986 |
|  | TNFRSF19 | TNF receptor superfamily member 19(TNFRSF19) | 2.38739 |
|  | TNFRSF21 | TNF receptor superfamily member 21(TNFRSF21) | 2.40158 |
|  | TNFSF13B | TNF superfamily member 13b(TNFSF13B) | -1.80866 |
|  | TNFSF15 | TNF superfamily member 15(TNFSF15) | -3.99867 |
|  | TNFSF4 | TNF superfamily member 4(TNFSF4) | 3.81215 |
|  | TNFSF9 | TNF superfamily member 9(TNFSF9) | -1.89731 |
| **PI3K-Akt signaling pathway** | CHRM2 | cholinergic receptor muscarinic 2(CHRM2) | 2.94501 |
|  | COL4A5 | collagen type IV alpha 5 chain(COL4A5) | -2.07434 |
|  | COMP | cartilage oligomeric matrix protein(COMP) | 1.81469 |
|  | DDIT4 | DNA damage inducible transcript 4(DDIT4) | 2.86687 |
|  | EFNA5 | ephrin A5(EFNA5) | -1.57785 |
|  | EIF4EBP1 | eukaryotic translation initiation factor 4E binding protein 1(EIF4EBP1) | 1.97126 |
|  | ERBB4 | erb-b2 receptor tyrosine kinase 4(ERBB4) | 2.75274 |
|  | F2R | coagulation factor II thrombin receptor(F2R) | -2.32079 |
|  | FGF16 | fibroblast growth factor 16(FGF16) | -2.11274 |
|  | FGF18 | fibroblast growth factor 18(FGF18) | -3.50232 |
|  | FGF9 | fibroblast growth factor 9(FGF9) | 1.92192 |
|  | FGFR2 | fibroblast growth factor receptor 2(FGFR2) | -2.90333 |
|  | GHR | growth hormone receptor(GHR) | -2.98434 |
|  | IGF2 | insulin like growth factor 2(IGF2) | -1.53061 |
|  | IL6 | interleukin 6(IL6) | 5.74887 |
|  | IL7 | interleukin 7(IL7) | -2.5209 |
|  | IL7R | interleukin 7 receptor(IL7R) | 2.14094 |
|  | ITGA11 | integrin subunit alpha 11(ITGA11) | 1.97574 |
|  | ITGA7 | integrin subunit alpha 7(ITGA7) | -4.05664 |
|  | ITGA8 | integrin subunit alpha 8(ITGA8) | -2.23313 |
|  | ITGB8 | integrin subunit beta 8(ITGB8) | 2.85069 |
|  | JAK3 | Janus kinase 3(JAK3) | 1.85748 |
|  | LAMA5 | laminin subunit alpha 5(LAMA5) | -4.31992 |
|  | LAMC3 | laminin subunit gamma 3(LAMC3) | -1.77034 |
|  | NTF3 | neurotrophin 3(NTF3) | -1.91661 |
|  | NTRK2 | neurotrophic receptor tyrosine kinase 2(NTRK2) | -5.13502 |
|  | PCK2 | phosphoenolpyruvate carboxykinase 2, mitochondrial(PCK2) | 3.03111 |
|  | PGF | placental growth factor(PGF) | 4.04587 |
|  | PIK3R1 | phosphoinositide-3-kinase regulatory subunit 1(PIK3R1) | -1.52064 |
|  | PIK3R3 | phosphoinositide-3-kinase regulatory subunit 3(PIK3R3) | -1.79428 |
|  | PPP2R2B | protein phosphatase 2 regulatory subunit Bbeta(PPP2R2B) | -2.98777 |
|  | PRLR | prolactin receptor(PRLR) | -2.16611 |
|  | THBS4 | thrombospondin 4(THBS4) | 2.51182 |

**Table S3 Ranked clusters of differentially expressed genes (DEG) network in CMT4B3 fibroblasts analyzed by MCODE**. To gain a better understanding of the physical relationships between DEGs, we used the Molecular Complex Detection (MCODE) Cytoscape plug-in, version 2.0.0, which identified the most important sub-modules of the network map. The criteria used for MCODE analysis were a degree cut-off of two, MCODE scores greater than five, a maximum depth of 100, a node score cut-off of 0.2, and a k-score of 2. Subsequently, we employed the Biological Networks Gene Ontology tool (BiNGO), version 3.0.3, to analyze and visually represent the biological processes of the identified sub-modules, with an FDR corrected P-value of less than 0.001.

| **Cluster** | **Score (Density*#Nodes)** | **Nodes** | **Edges** | **Node IDs** | **GO Biological Processes P<0.001*** |
| --- | --- | --- | --- | --- | --- |
| 1 | 9,5 | 10 | 43 | KCNB1, KCNS2, KCNA1, KCNA4, KCNS3, KCND2, KCNQ5, KCNQ3, KCNE4, KCNG1 | ion transport, transmembrane transport, potassium ion transport, establishment of localization, transport localization, monovalent inorganic cation transport, metal ion transport, cation transport. |
| 2 | 8,9 | 10 | 40 | OAS1, HLA-B, OAS2, HLA-C, MX1, OAS3, RSAD2, USP18, MX2, LGALS3BP | immune response, immune system process, defense response, response to stimulus, antigen processing and presentation of peptide antigen via MHC class I, response to virus, multi-organism process |
| 3 | 7.3 | 25 | 85 | WNT16, COL21A1, ADAMTS9, ADAMTS15, WNT11, COL14A1, FRZB, ADAMTSL3, NOTCH3, ADAMTS7, ADAMTS8, COL4A5, ADAMTS3, ADAMTSL4, COLGALT2, PTCH1, ADAMTS6, COL12A1, P4HA3, COL11A1, COL8A1, SFRP2, WISP1, DKK1, COL15A1 | collagen fibril organization, negative regulation of Wnt receptor signaling pathway, negative regulation of canonical Wnt receptor signaling pathway, regulation of Wnt receptor signaling pathway |
| 4 | 5.6 | 6 | 14 | FABP3, PDK4, CPT1C, SCD, DGAT2, PCK2 | small molecule metabolic process, monocarboxylic acid metabolic process, alcohol metabolic process, oxoacid metabolic process, carboxylic acid metabolic process, cellular lipid metabolic process, organic acid metabolic process, cellular ketone metabolic process, fatty acid metabolic process |
| 5 | 5,2 | 21 | 52 | GRIN2A, RSPO1, MCAM, RSPO3, GHR, TGFB2, BMP4, PTGS2, PRLR, BMP2  JAG1, AXIN2, ACAN, FOS, WLS, PIK3R1  TGFBI, WNT5A, ERBB4, PGF, BGN | positive regulation of endothelial cell proliferation, pathway-restricted SMAD protein phosphorylation, positive regulation of cartilage development, response to vitamin, Wnt receptor signaling pathway, mesenchymal cell development, mesenchymal cell differentiation, response to mechanical stimulus |

***** False Discovery Rate (FDR) P<0.001 with BiNGO.

**Table S4 Primary antibodies for western blot and immunofluorescence analysis.**

| **Antibody** | **Species** | **Diluition for WB/IF** | **Company** | **Catalog number** |
| --- | --- | --- | --- | --- |
| **OPA1** | **mouse** | **1:500 (WB)** | **BD Tranduction Laboratories** | **612606** |
| **MFN2** | **mouse** | **1:1000 (WB)** | **Abnova** | **H00009927-M03** |
| **p-53** | **mouse** | **1:1000 (WB)** | **Santa Cruz Biotechnology** | **Sc-126** |
| **p-21** | **rabbit** | **1:500 (WB)** | **Santa Cruz Biotechnology** | **Sc-397** |
| **mtTFA** | **mouse** | **1:1000 (WB)** | **Novus Biologicals** | **18G102B2E11** |
| **TFEB** | **rabbit** | **1:750 (WB)** | **Abcam** | **Ab113372** |
| **ND4** | **rabbit** | **1:1000 (WB)** | **Santa Cruz Biotechnology** | **Sc-20499-R** |
| **NDUFS1** | **rabbit** | **1:500 (WB)** | **Santa Cruz Biotechnology** | **Sc-99232** |
| **SDHA** | **rabbit** | **1:500 (WB)** | **Cell Signaling Technology** | **5939S** |
| **COX IV** | **rabbit** | **1:1000 (WB)** | **Cell Signaling Technology** | **4844S** |
| **GAPDH** | **mouse** | **1:15000 (WB)** | **ProteinTech** | **60004-1** |
| **Phospho-AKT Thr308** | **rabbit** | **1:1000 (WB)** | **Cell Signaling Technology** | **13038** |
| **Phospho-AKT Ser473** | **rabbit** | **1:2000 (WB)** | **Cell Signaling Technology** | **4060** |
| **AKT tot** | **mouse** | **1:2000 (WB)** | **Cell Signaling Technology** | **2920** |
| **ATP5FB1** | **rabbit** | **1:3000 (WB)** | **ProteinTech** | **17247-1-AP** |
| **TOMM20** | **mouse** | **1:1000 (WB)** | **Abcam** | **Ab56783** |
| **VDAC1/Porin** | **mouse** | **1:500 (IF)** | **Abcam** | **Ab186321** |
